# ^1^H-NMR Guided Isolation of Bioactive Compounds from Species of the Genus *Piper*

**DOI:** 10.3390/molecules30092020

**Published:** 2025-04-30

**Authors:** Celso R. Oliveira, Megan J. Burroughs, Lora A. Richards, Lee A. Dyer, Federico Urbano-Muñoz, Camryn Lee, Megan Warner, Craig D. Dodson, Ian S. Wallace, Christopher S. Jeffrey

**Affiliations:** 1 Department of Forestry and Wildlife Ecology, University of Wisconsin-Madison, Madison, WI 53706, USA; celso.chemistry@gmail.com; 2 Department of Chemistry and Biochemistry, University of Missouri-St. Louis, 1 University Blvd. 316 Benton Hall, St. Louis, MO 63121, USA; mbtdk@umsl.edu; 3 Hitchcock Center for Chemical-Ecology, Department of Biology, University of Nevada-Reno, Reno, NV 89557, USA; lorar@unr.edu (L.A.R.); nolaclimber@gmail.com (L.A.D.); 4 Department of Biochemistry and Molecular Biology, University of Nevada-Reno, Reno, NV 89557, USA; furbanomunoz@nevada.unr.edu (F.U.-M.); camrynl@nevada.unr.edu (C.L.); meganwarner500@gmail.com (M.W.); 5 Hitchcock Center for Chemical-Ecology, Department of Chemistry, University of Nevada-Reno, Reno, NV 89557, USA; craigd@unr.edu; 6 Complex Carbohydrate Research Center, University of Georgia, Athens, GA 30606, USA

**Keywords:** ^1^H NMR-based metabolomics, antimicrobial compounds, bioactive natural products isolation, WGCNA

## Abstract

The discovery of bioactive natural products is often challenged by the complexity of isolating and characterizing active compounds within diverse mixtures. Previously, we introduced a ^1^H NMR-based weighted gene correlation network analysis (WGCNA) approach to identify spectral features linked to growth inhibitory activity of *Piper* (*Piperaceae*) leaf extracts against model plant, fungal, and bacterial organisms. This method enabled us to prioritize specific spectral features linked to bioactivity, offering a targeted approach to natural product discovery. In this study, we validate the predictive capacity of the WGCNA by isolating the compounds responsible for the bioactivity-associated resonances and confirming their antifungal efficacy. Using growth inhibition assays, we verified that the isolated compounds, including three novel antifungal agents, exhibited significant bioactivity. Notably, one of these compounds contains a rare imidazolium heterocyclic motif, marking a new structural class in *Piper*. These findings substantiate the ^1^H NMR-based WGCNA as a reliable tool for identifying structural types associated with biological activity, streamlining the process of discovering bioactive natural products in complex extracts.

## 1. Introduction

Natural products have long been a cornerstone of drug discovery, providing structurally diverse bioactive compounds [1]. However, traditional bioassay-guided fractionation can be slow and inefficient, often leading to the repeated isolation of known compounds. Advances in metabolomics and network-based analytical approaches now offer more targeted strategies for identifying novel bioactive molecules and include the use of NMR-based metabolomic approaches [2,3,4,5,6]. Previously, we introduced a ^1^H NMR-based weighted gene co-expression network analysis (WGCNA) approach to identify spectral features linked to antifungal activity in *Piper* (*Piperaceae*) extracts. This method enabled us to correlate specific ^1^H NMR resonances with bioactivity, effectively prioritizing candidate compounds for isolation [7]. While WGCNA demonstrated strong predictive power, direct validation through isolation, structural characterization, and verification of the bioactivity of purified target compounds was necessary.

The *Piper* genus (*Piperacace*), comprising over 2500 species with a pantropical distribution, has been widely studied in chemical ecology and bioactive molecule discovery due to its rich phytochemical diversity and role in plant defense mechanisms [8,9,10,11,12]. Our research has focused on *Piper* as a model for understanding chemically mediated ecological interactions, particularly in plant–pathogen and plant–herbivore relationships [13,14,15,16,17,18]. Known for producing biologically active secondary metabolites, including antifungal amides and phenylpropanoids, *Piper* serves as an ideal system for studying how secondary metabolites contribute to ecological resilience and potential applications.

In this study, we aimed to evaluate the predictive power of ^1^H NMR-based WGCNA by isolating and structurally characterizing bioactive compounds corresponding to antifungal activity-associated spectral features. By targeting resonances affiliated with the specific modules identified in the WGCNA analysis, we successfully isolated three antifungal compounds, including a rare imidazolium-containing natural product. Growth-inhibition assays confirmed their antifungal efficacy, directly linking the predicted spectral features to functional bioactive molecules. These findings substantiate WGCNA as a viable method for identifying bioactive compounds in complex natural product mixtures, streamlining the discovery of biologically relevant metabolites.

## 2. Results

### 2.1. Target Identification for Isolation, Characterization and Biological Verification

**Figure 1:**
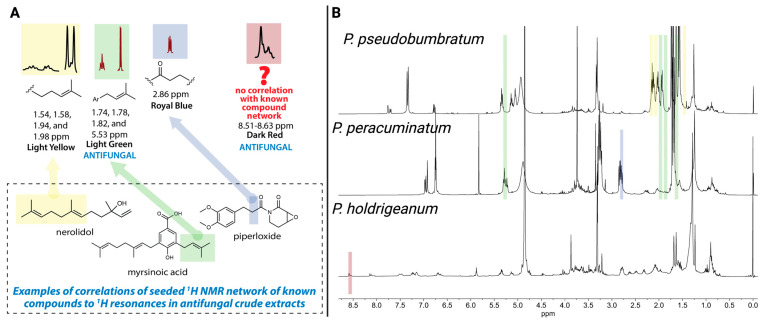
(**A**) Identification of target ^1^H NMR resonances and predicted structural motifs based on previous studies [7,19]. (**B**) Crude ^1^H NMR spectra of methanolic extracts from three *Piper* species with confirmed antifungal activity. Colored module overlays (light yellow, light green, royal blue, and dark red) indicate chemical shift regions correlated with specific extracts. The dark red module (δ_H_ 8.61–8.53); *P. holdridgeanum*) and light green module (δ_H_ 5.33, 1.82, 1.78, 1.74); *P.dobumbratum* and *P. peracuminatum*) were significantly associated with antifungal activity and were targeted for isolation. Created in BioRender. Jeffrey, C. (2025) https://BioRender.com/9k9pvls.

In our previous study of ^1^H NMR spectra from methanolic extracts of 30 *Piper* species, we used network analysis to identify spectral modules correlated with antifungal activity [7,19]. Two modules showed significant correlations with *Saccharomyces cerevisiae* inhibition: a light green module linked to *P. peracuminatum* and *P. pseudobumbratum* and a dark red module associated with *P. holdridgeanum*. Structural annotation of these modules, aided by a library of known *Piper* compounds, indicated that the light green module matched spectral features of prenylated aryl acids such as myrsinoic acid, reflecting the dominant prenylated phenol resonances present in both *P. peracuminatum* and *P. pseudobumbratum*; however, the specific structures of the active compounds remained unknown (Figure 1). The dark red module contained an unusual signal (δ_H_ 8.53–8.62) with no match to known compounds, suggesting a novel scaffold in *P. holdridgeanum* that was not represented by the group of known *Piper* compounds (Figure 1). Importantly, this signal originated from a minor component of the extract, unlike the other two active extracts. These spectral insights guided our targeted isolation of antifungal compounds from these three species, resulting in the isolation of the active compounds that contain these resonances and validating the predictive power of the ^1^H NMR network-based approach.

### 2.2. Compound Isolation Guided by Targeted 1H NMR Resonances

To isolate the compounds that correlated to the bioactivity, we adopted a general isolation protocol that minimized the number of fractions and the necessity of repeated bioactivity screening by tracking the target resonances through ^1^H NMR analysis of semi-pure and pure fractions. The crude methanolic extracts were pre-fractionated using a C18 column eluted with 50–100% *v*/*v* acetone/H_2_O. Fractions were analyzed by ^1^H NMR, and those containing the target resonances were then prioritized for further purification via reverse-phase preparatory chromatography using C18.

### 2.3. Structure Determination of Target Compounds

**Figure 2:**
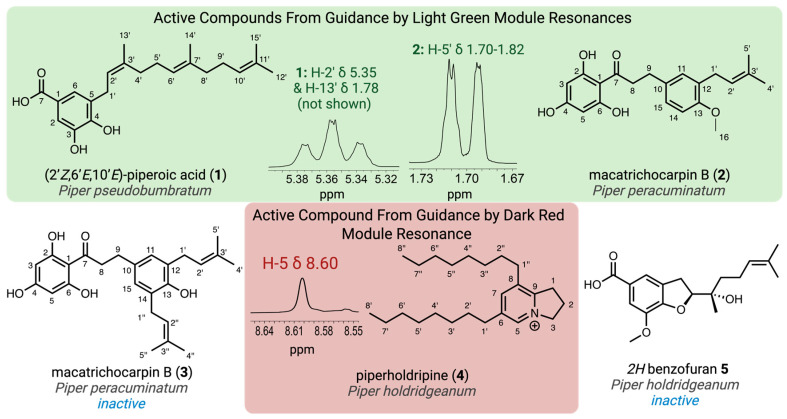
Structures isolated from the crude extracts using the ^1^H NMR targeted approach along with the specific resonances that guided the isolation from the light green and dark red modules. Created in BioRender. Jeffrey, C. (2025) https://BioRender.com/tou5huv.

The isolated compounds were screened against *S. cereviseae* to confirm inhibitory activity and subsequently analyzed using various 2D NMR techniques for structural characterization. ^1^H NMR guided isolation from *P. pseudobumbratum*, *P. peracuminatum*, and *P. holdridgeanum* resulted in the purification of three compounds (**1**, **2**, and **4**, Figure 2) that contained the target resonances and demonstrated antifungal activity consistent with the inhibitory activity of the crude extracts. During the process of the ^1^H NMR targeted isolation, additional major compounds were purified and characterized from the focal species; however, they did not demonstrate antifungal activity (**3** and **5**, Figure 2).

Initial fractionation of the *P. pseudobumbratum* extract yielded the target compound at high concentration from the chromatographic fraction eluted with 50% acetone/water on C18. Further purification of this extract provided 35 mg of compound **1** with an ESI-HRMS *m*/*z* 357.2009 (calculated for C_22_H_30_O_4_, 357.2071 [M − H]^−^), which displayed the signature resonances from module light green. Analysis of the ^1^H NMR data suggested the presence of a 1,2,3,5-tetrasubstitued aromatic ring with a single farnesyl substituent, which was characterized by the presence of three vinylic resonances (*δ*_H_ 5.07, 5.16 and 5.36) and the single benzylic methylene (*δ*_H_ 3.33) (Table 1). From the ^13^C NMR data, we concluded that one of the ring substituents was a carboxylic acid (*δ*_C_ 170.7 ppm), with the other two assigned as phenols given the presence of two oxygenated aryl carbons (*δ*_C_ 149.4 and 145.4). Finally, we concluded that both aromatic protons H-2 and H-6 were *ortho* to the carboxylic acid since they displayed ^1^H{^13^C} HMBC correlations with this quaternary carbon (C-7) and one of the hydroxylated carbons (C-4). The observed chemical shift values at *δ*_H_ 7.32 and 7.35 and *meta* coupling constant (*J* = 2 Hz) were consistent with this substitution pattern. NOESY data confirmed the configuration of the alkene groups, and the final structure was determined as (2’*Z*,6’*E*,10’*E*)-piperoic acid (**1**), which is a new isomeric form of the equivalent (2’*E*,6’*Z*,10’*E*) compound isolated from *P. auritum* [20].

Solid-phase partitioning of the *P. peracuminatum* extract led to the recovery of the target peaks in the fractions eluted with 50% and 60% acetone/water. These fractions were pooled based on the targeted resonances for purification, and the resulting collections yielded two novel compounds–**2** (20 mg) and **3** (16 mg)–with proximal eluting times that contained the target resonances. Compound **2** resulted in an ESIHRMS *m*/*z* 355.1560 (calculated for C_21_H_24_O_5_, 355.1551 [M − H]^−^), and compound **3** yielded an ESIHRMS *m*/*z* 409.2030 (calculated for C_25_H_30_O_5_, 409.2020 [M − H]^−^). Both compounds also had the signature peak from module royal blue (*δ*_H_ 2.86, H-9), which was confirmed to be part of a propenone moiety based on its ^1^H{^1^H} COSY correlations with the methylene resonance at ~*δ*_H_ 3.25 (H-8) and on the ^1^H{^13^C} HMBC peaks of both methylene resonances with the ketone resonance at *δ*_C_ 205.9 (C-7) (Table 2). The peak from H-9 was present at an integration ratio of 1:1 with the vinylic resonance (~*δ*_H_ 3.25, H-2’) in compound **3**, which led us to the conclusion that this compound possessed two equivalent prenyl groups (Table 3). The most distinctive proton peak patterns for the two compounds were verified in the aromatic region, where compound **2** displayed three diagnostic aryl-H resonances for a 1,2,4-trisubstituted ring while **3** contained a singlet indicative of a symmetric 1,2,3,4-tetrasubstituted ring. We confirmed from the ^1^H{^13^C} HMBC correlations between these aromatic protons and the carbons C-9 and C-1’ that the prenyl groups were implicated in the symmetry of the aromatic ring of **3** and that in both compounds, the propenone moiety was attached to the ring through the β-carbonyl carbon and *meta* position relative to the prenyl groups. The presence of an oxygenated aryl carbon (C-13) in both compounds led to the conclusion that the remaining substituent was a hydroxyl group in compound **3** and a methoxy group in **2** (*δ*_H_ 3.78, H-16). Finally, we identified the singlet at *δ*_H_ 5.86 as being part of an acylphloroglucinol aromatic moiety, which was connected to the propenone linker through the carbonyl carbon (^1^H{^13^C} HMBC, C-7, and H-3/5), thus defining the structures of macatrichocarpin B (**2**), which was previously isolated from *Macaranga trichocarpa*, and its 14-prenylated derivative (**3**) [21].

**Figure 3:**
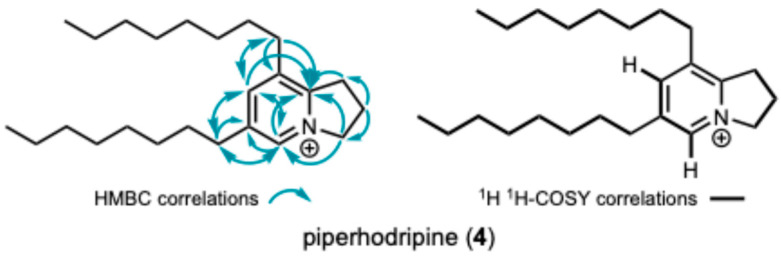
Key ^1^H, ^1^H COSY, and ^1^H, ^13^C HMBC correlations leading to the assignment of the structure of piperholdripine (**4**).

Initial attempts to purify the bioactive component(s) from *P. holdrigeanum* were met with failure, only resulting in the isolation of a methylated carboxylic acid derivative of a 2*H*-benzofuran previously isolated from *Waltheria indica* (**5**; see supporting information for the details of the structure determination) that did not contain the targeted resonances from module dark red and demonstrated no antifungal activity against *S. cerevisiae* [22]. We found that silica gel chromatography was not amenable to the isolation of the compound containing the targeted resonances and speculated that the lack of a strong chromophore also limited the application of traditional isolation methods using reverse-phase media with UV detection. By employing reverse-phase prep-HPLC with MSD detection to partition the crude extract, we recovered 10 fractions containing varying concentrations of the compound(s) exhibiting the target resonances (*δ*_H_ 8.61). All these fractions exhibited potent antifungal activity against *S. cerevisiae* and contained a component with *m*/*z* 344.3320 corresponding to C_24_H_42_N^+^ (calculated for 344.3312 [M]^+^). Further purification of the pooled fractions led to a highly enriched fraction of the target compound containing the singlet (*δ*_H_ 8.61) identified by module dark red along with a propenyl system [*δ*_H_ 4.83 (t), 2.50 (pent), and 3.47 (t), coupled via COSY] and two long-chain aliphatic substituents. The diagnostic ^1^H NMR resonances were assigned to the 5 and 7 positions of the indolizinium core, and ^13^C NMR analysis further demonstrated the presence of five aromatic carbon resonances (Table 4). HMBC analysis confirmed the connectivity of the substituents to the aromatic ring, which supported the assignment of the novel C6,C8*-bis*-octyl substituted 2,3-dihydro-1*H*-indolizidinium alkaloid, piperholdripine (**4**), as the principal bioactive compound from *Piper holdridgeanum* and represents a plant-derived indolizinium compound that demonstrates antimicrobial activity (Figure 3) [23,24,25,26].

**Scheme 1:**
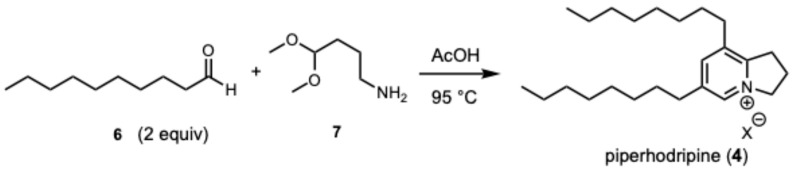
A confirmatory synthesis of piperholdripine (**4**) from decanal and 4-aminobutanal dimethyl acetal.

To verify the structure of piperholdripine (**3**), we pursued a confirmatory synthesis using methods reported by Snider [27]. Heating a solution of decanal (**6**) with 4-aminobutanal dimethyl acetal (**7**) in acetic acid at 80 °C provided piperholdripine (**4**) in 17% yield after isolation using flash reverse-phase chromatography (C-18, acetonitrile/water) which was spectroscopically found to be identical to the isolated compound (Scheme 1).

### 2.4. Bioactivity of the Isolated Compounds

**Figure 4:**
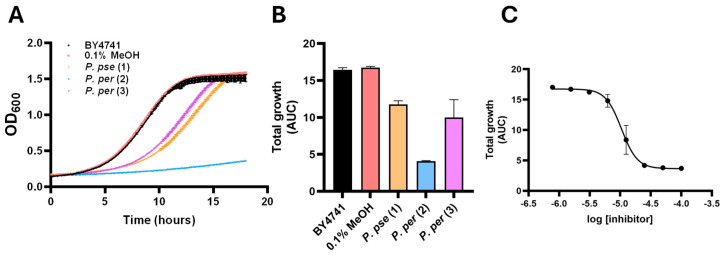
Antifungal activity of P. *pericuminatum* and *pseudobumbaratum* compounds. Purified compounds were assayed against *S. cerevisiae* BY4741 as described in Materials and Methods at a concentration of 100 µM. *S. cerevisiae* treated with 0.1% MeOH served as a solvent control. (**A**) Representative growth curves of *S. cerevisiae* BY4741 treated 100 µM (2’*Z*,6’*E*,10’*E*)-piperoic acid (**1**) (orange), macatrichocarpin (**2**) (blue), or 5-prenyl macatrichocarpin (**3**) (purple) compared to 0.1% MeOH (red) and untreated (black) growth curves. Error bars represent SEM (*n* = 3). (**B**) AUC values of integrated growth curves are shown for similar experiments. Error bars represent SEM (*n* = 3). (**C**) Macatrichocarpin (**2**) was assayed at decreasing concentrations to establish a dose–response relationship. Error bars represent SEM (*n* = 3).

The purified compounds were subjected to *S. cerevisiae* growth inhibition assays to investigate their antifungal properties. We first assayed the isolated compounds from *P. pseudobubratum* and *P. peracuminatum* at a standard concentration of 100 µM in *S. cerevisiae* growth inhibition assays as described in the supporting information (Figure 4A,B) [28]. (2’*Z*,6’*E*,10’*E*)-Piperoic acid (**1**) from *P. pseudobumbratum* inhibited *S. cerevisiae* growth by 25% compared to untreated or solvent controls. In contrast, macatrichocarpin B (**2**) inhibited *S. cerevisiae* growth by 75%, and cells treated with this compound exhibited markedly extended lag phase growth compared to untreated cells. Dose–response analysis of cells treated with macatrichocarpin B (**2**) indicated an IC_50_ of 10.5 µM (Figure 4C). 14-prenyl macatrichocarpin B (**3**) was less active at 100 µM compared to macatrichocarpin B (**2**), displaying only 40% growth inhibition compared to untreated controls. These results suggest that the prenyl substitution captured by module light green is a determining structural feature for compound activity in the investigated extracts, which is further supported by reports that identify prenylated benzoic acid derivatives with antifungal activity across several species of *Piper* [20,29,30]. It is noteworthy that although this structural feature was correlated with increased inhibitory activities of compounds **1** and **2**, the presence of a second prenyl unit in 5-prenyl macatrichocarpin B (**3**) resulted in significantly reduced potency, suggesting a structural specificity affiliated with *S. cerevisiae* inhibition. Moreover, the enhanced inhibitory effect of macatrichocarpin B (**2**) was associated with another module (royal blue) that represented part of the dihydrochalcone motif, which is also present in antifungal compounds isolated from *P. mollicomum* and *P. aduncum*, and that can be postulated as an important structural determinant for yeast inhibition. Conversely, the module that was most strongly associated with *P. pseudobumbratum* (light yellow) was not correlated with inhibitory activity as it represented structural features of an elongated farnesyl group that were not as determining for inhibitory activity [31,32,33]. This is a minus sign or at least this is the minus sign that graphpad prism uses and can’t really be changed in the program.

**Figure 5:**
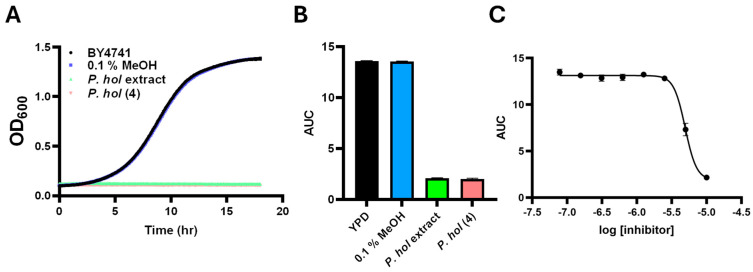
Growth inhibition analysis of purified piperholdripine (4): *S. cerevisiae* BY4741 was grown in YPD medium supplemented with 80 µg/mL *P. holdrigeanum* extract, 100 µM purified compound 4, or 0.1% MeOH as a negative control for 18 h at 30°. (**A**) Representative growth curves of samples are shown. (**B**) Calculated and plotted growth curves. Error bars represent SEM (*n* = 3 biological replicates, 9 technical replicates). (**C**) Dose–response curves of *S. cerevisiae* cultures subjected to decreasing concentrations of piperholdripine (**4**).

The antifungal activity of *P. holdridgeanum* extracts (Figure 5A,B) revealed an inhibitory effect comparable to that observed for *P. peracuminatum* but originating from a compound that was present at a considerably lower concentration according to the target resonances depicted by the network analysis. Piperholdripine (**4**), originating from isolation and synthesis, demonstrated strong antifungal activity, inhibiting *S. cerevisiae* growth by greater than 80% compared to untreated and solvent controls (Figure 5A,B). Dose–response analysis of piperholdripine (**4**) growth inhibition activity indicated an IC_50_ of 4.9 µM (Figure 5C). This compound did not inhibit the growth of the model bacterium *Escherichia coli*, demonstrating growth inhibition selectivity toward *S. cerevisiae*.

## 3. Discussion

In summary, we verified that the ^1^H NMR network led to the identification of compounds (including two new molecules), which were found to be responsible for the bioactivity observed for the crude leaf extracts (Table 5). The light green module directed the isolation toward prenylated phenols and led to the isolation of *iso*-piperoic acid (**1**) and macatrichocarpin B (**2**), which both demonstrated inhibitory activity against *S. cerevisiae*. The inhibitory activity of *iso*-piperoic acid (**1**) was the lowest among the isolated compounds and lower than that of the crude extract (28.5% vs. 63.2% inhibition). However, direct quantitative comparison is challenging due to the unknown concentration of active component(s) within the crude mixture. Furthermore, potential synergistic effects or contributions from unidentified minor constituents may enhance the bioactivity of the crude extract, and further investigation is needed to fully assess these factors. The activity of macatrichocarpin B (**2**) was found to inhibit *S. cerevisiae* (IC_50_ = 10.9 μM) at a much closer level to the crude extract (75.3 vs. 98.1% inhibition). In addition to the light green module resonances, the dark red module that was represented by a resonance (*δ*_H_ 8.6) in the crude extract of *Piper holdridgeanum* and correlated with inhibitory activity against *S. cerevisiae* led to the isolation and characterization of piperholdripine (**4**). Piperholdripine (**4**) is an indolizinium natural product that represents a new compound that has not been observed across the *Piper* genus. The pure compound demonstrated the most potent inhibitory activity (IC_50_ = 4.9 μM) against *S. cerevisiae* in this study and close to that observed for the crude extract (85.3 vs. 95.8% inhibition).

Given the validation of the ^1^H NMR approach, we predict that the evaluation of a larger library of *Piper* extracts and integration of a larger set of known compounds using this methodology may further elucidate the structural effects (e.g., elongation and functionalization of the prenyl chain) in modulating the antifungal activities of these compounds and to guide the isolation of novel molecular structures associated with biological activity. Complementarily, the application of this approach with yeast deletion collections could provide a powerful method to identify the specific mechanisms of action of a compound [34]. Gene knockouts that result in altered drug resistance may offer direct clues to structure-cellular target affiliations and thus help to discern whether varying levels of inhibition result from differentiated compound-specificity towards a common target or if these compounds target distinct cellular processes. The refined structural information obtained from the chemical modules can then be evaluated against susceptible/resistant phenotypes to highlight the role of specific molecule motifs in dictating biological activity and be used to guide the isolation of bioactive constituents of a mixture based upon ^1^H NMR resonances.

## 4. Materials and Methods

### 4.1. Compound Isolation

For the isolation of target compounds, we sequentially and extensively extracted 6 g of oven-dried leaf material with *n*-hexane, acetone, and methanol at room temperature under mechanical agitation [7]. For all three focal *Piper* species, the acetone fractions displayed the target peaks upon ^1^H NMR analysis, so we pre-fractionated 200 mg of these extracts using preparative reverse-phase medium pressure liquid chromatography (RP-MPLC; 10 g column, FC-C18 60 μm, 2.5 cm × 8 cm), eluted with acetone/H_2_O gradient starting at 50% *v*/*v* and increasing by discrete increments of 10% *v*/*v*. The stationary phase was pre-conditioned with 50% acetone/H_2_O, and then the extracts were suspended in approximately 1 mL of mobile phase and injected into the column. Two samples of 15 mL were collected for each eluent mixture, then dried under reduced pressure and analyzed by ^1^H NMR. Fractions containing the target resonances were then further purified using RP-MPLC (5 g, FC-C18 60 μm, 1.5 cm × 5 cm, Yamazen), eluting with an appropriate linear gradient of acetone/H_2_O, and monitored by thin layer chromatography and UV absorption at 256 nm. Compound **1** was recovered in the 50% fractions of the *P. pseudobumbratum* extract and purified using the aforementioned chromatographic setup with a 30–60% *v*/*v* acetone/H_2_O gradient. Compounds **2** and **3** were obtained as a co-eluting mixture from the second 50% and the first 60% fractions of the *P. peracuminatum* extract and were purified in a similar manner using a 40–70% acetone/H_2_O gradient. The identity and purity of the isolated compounds were confirmed by ^1^H NMR analysis.

Extraction of dried leaves of *Piper holdrigeanum* with methanol provided 5.1 g of crude extract. *Piper holdripine* was isolated using preparative HPLC chromatographic separation, which was performed using an Agilent Infinity II Preparative LC system paired with a 6140 quadrupole MSD and variable wavelength detection. The HPLC with a gradient elution method of water and acetonitrile containing 0.1% formic acid using an Agilent InfinityLab Poroshell 120 4 SB-C18 column (21.1 × 150 mm, 25 mL/min) and a gradient starting at 70:30 water/acetonitrile and linearly increasing to 17:83 water/acetonitrile over 20 min followed by step increase to 100% acetonitrile for 10 min Agilent. This method resulted in 10.5 mg of a partially purified fraction containing the target compound. The resulting sample was further purified using the Agilent, a semi-preparative HPLC column (Agilent 5 Prep-C18 250x10mm, 8mL/min flow rate) using the identical water/acetonitrile gradient previously described, resulting in the purified sample of piperholdripine (**4**), which eluted as a broad peak eluting at approximately 13.2 min (0.8 mg, 344.4 m/z).

### 4.2. Spectroscopic Analysis

Purified compounds were resuspended in methanol-*d*_4_ containing 0.03% TMS to an approximate concentration of 10 mg/mL and subjected to complete structural characterization. One-dimensional and two-dimensional NMR analysis was conducted on a Varian 400-MR or a Varian 500 MHz spectrometer using the following number of transients: ^1^H: 128; 13C: 15,000; gCOSY: 4 × 128; gHSQC: 4 × 256; and gHMBC: 8 × 512. Optimal pulse widths (pw90), delay, and acquisition times were experimentally determined for each compound. In cases where the solvent residual peaks compromised the characterization of the compounds, samples were also prepared and analyzed using acetonitrile-*d*_3_ to resolve the assignments when necessary. High-resolution mass spectra for compounds **2**–**4** were performed on an Agilent 1200 series HPLC coupled to an Agilent 6230B TOFMS. The HPLC was run in a flow injection using an isocratic mobile phase consisting of 0.1% formic acid in acetonitrile with a flow rate of 0.4 mL/min. An amount of 1 μL of sample was injected into the mobile phase stream, and the mass spectrum was collected for 1 min. The sample was analyzed in position ion mode on the TOMFS with the following source conditions: 325 °C temperature; nitrogen gas flow of 5 l/min at a pressure of 20 psi; V*cap* = 3500 V; and a fragmentor set at 175 V, with a skimmer voltage of 65 V, and an Oct RF V*pp* of 750 V. HRMS was recorded for compound **1** by injecting (10.00 µL) on an Agilent 1290 Infinity II UPLC coupled to an Agilent 6560 Ion Mobility Quadrupole Time-of-Flight (IM-QTOF) mass spectrometer via a Jet Stream electrospray ionization source (ESI-TOF; gas temperature: 300 °C; flow: 11 L/m; nebulizer pressure: 35 psig; sheath gas temp: 300 °C; sheath gas flow: 12 L/m; VCap: 3500 V; nozzle voltage: 500 V; fragmentor: 350 V; skimmer: 65 V; octopole: 750 V; ion polarity: negative). All organic solvents and mobile phase modifiers were Fisher Optima grade. Mobile phase solvents consisted of 18 MΩ water (solvent A) and 99% acetonitrile (aq, solvent B), both modified by adding 0.1% formic acid.

**(2’*Z*,6’*E*,10’*E*)-piperoic acid (1): **^1^H NMR (400 MHz, CDCl_3_): δ 7.32 (d, *J* = 2.0 Hz, 1H), 7.35 (dt, *J* = 2.0, 0.5 Hz, 1H), 3.33 (br d, *J* = 7.1 Hz, 2H), 5.36 (br t, *J* = 7.4 Hz, 1H), 2.21–2.15 (m, 2H), 2.16–2.09 (m, 2H), 5.16 (br t, *J* = 7.4 Hz, 1H), 1.98–1.92 (m, 2H), 2.08–2.01 (m, 2H), 5.07 (m, 1H), 1.65 (br s, 3H), 1.75 (br q, *J* = 1.2 Hz, 3H), 1.60 (br s, 3H), 1.58 (br s, 3H); ^13^C NMR (101 MHz, CDCl_3_): δ 122.2, 115.1, 145.4, 149.4, 137.3, 124.1, 170.7, 28.8, 124.0, 129.2, 32.9, 27.8, 125.3, 136.2, 40.8, 27.8, 125.5, 132.0, 25.9, 23.8, 16.1, and 17.7. HRMS (ESI negative) m/z [M − H]^−^ calcd for C_22_H_30_O_4_, 357.2071; found 357.2079.

**macatrichocarpin B (2): **^1^H NMR (400 MHz, CDCl_3_): δ 7.00 (dd, *J* = 8.2, 2.3 Hz, 1H), 6.99 (d, *J* = 2.0 Hz, 1H), 6.79 (d, *J* = 8.3 Hz, 1H), 5.86 (s, 2H), 5.25 (m, 1H), 3.78 (s, 3H), 3.27–3.24 (m, 2H), 3.25 (br d, *J* = 7.4 Hz, 2H), 2.85 (dd, *J* = 8.2, 6.7 Hz, 2H), 1.71 (br s, 3H), 1.69 (br s, 3H); ^13^C NMR (101 MHz, CDCl_3_): δ 205.9, 165.2, 164.9, 156.6, 134.7, 133.0, 130.6, 130.5, 127.7, 123.7, 111.6, 105.3, 95.9, 56.1, 46.7, 30.5, 29.3, 25.9, and 17.8. HRMS (ESI negative) m/z: [M − H]^−^ calcd for C_21_H_24_O_5_, 355.1551;found 355.1560.

**14-prenylmacatrichocarpin B (3):**^1^H NMR (400 MHz, CDCl_3_): 7.35 (dt, *J* = 2.0, 0.5 Hz, 1H), 7.32 (d, *J* = 2.0 Hz, 1H), 5.36 (br t, *J* = 7.4 Hz, 1H), 5.16 (br t, *J* = 7.4 Hz, 1H), 5.07 (m, 1H), 3.33 (br d, *J* = 7.1 Hz, 2H), 2.21–2.15 (m, 2H), 2.16–2.09 (m, 2H), 2.08–2.01 (m, 2H), 1.98–1.92 (m, 2H), 1.75 (br q, *J* = 1.2 Hz, 3H), 1.65 (br s, 3H), 1.60 (br s, 3H), 1.58 (br s, 3H); ^13^C NMR (101 MHz, CDCl_3_): δ 170.7, 149.4, 145.4, 137.3, 136.2, 132.0, 129.2, 125.5, 125.3, 124.1, 124.0, 122.2, 115.1, 40.8, 32.9, 28.8, 27.8, 27.8, 25.9, 23.8, 17.7, and 16.1. HRMS (ESI negative) m/z: [M − H]^−^ calcd for C_25_H_30_O_5_, 409.2020; found 409.2030.

**Piperholdripine (4): **^1^H NMR (500 MHz, CDCl_3_): δ 8.60 (br s, 1H), 8.17 (br s, 1H), 4.83 (br t, *J* = 7.8 Hz, 2H), 3.47 (br t, *J* = 7.8 Hz, 2H), 2.80 (br t, *J* = 7.3 Hz, 4H), 2.50 (br pent, *J* = 7.8 Hz, 2H), 1.66–1.72 (m, 4H), 1.34–1.44 (m, 8H), 1.26–1.36 (m, 12H), 0.90 (m, 6H); ^13^C NMR (125 MHz, CDCl_3_): δ 156.6, 145.9, 142.9, 140.7, 138.8, 60.5, 33.2, 33.0, 32.7, 31.7, 31.6, 30.2, 30.1, 23.7, 22.1, 14.4. HRMS (ESI positive) m/z: [M]^+^ calcd for C_24_H_42_N^+^; found 344.3320.

### 4.3. Network Analysis

Network analysis of the crude ^1^H NMR spectra was performed according to previously reported methodology [7,19]. To facilitate the characterization of bioactive molecular targets from the crude extracts, we seeded the network analysis with spectra collected from a library of prepared mixtures of known compounds that broadly represent metabolite classes encountered in plant extracts. After the modules were calculated for the combined set of mixtures and extracts, we calculated the Pearson correlations between module eigenvalues and bioassay data. We then processed a parallel analysis to assign module correlations with compound concentrations from the mixtures. By comparing these results, we were then able to estimate the structural elements associated with the bioactive targets present in the crude extracts. Moreover, by referring to the modules that demonstrated a significant correlation with bioactivity, we identified diagnostic peaks in the ^1^H-NMR spectrum that were used as a guide for the isolation of the bioactive compounds.

### 4.4. Synthesis of Piperholdripine

Decanal (2.5 mL, 15.0 mmol, 2.0 equiv) was added to a solution of 4-aminobutanal dimethyl acetal (1.2 mL, 7.5 mmol, 1.0 equiv) in acetic acid (30 mL, 0.25 M). The solution was heated to 95 °C and stirred for 48 hr. After cooling, the reaction solution was made basic (pH~12) with 10M NaOH (70 mL), and the aqueous layer was washed with hexanes and extracted with chloroform. The combined chloroform layers were dried over MgSO_4_, filtered, and the solvent was evaporated under reduced pressure. Purification using solid-phase extraction on C18 (42g SepPack) with step elution with H_2_O in CH_3_CN (100%,75%, 50%, 0%) provided piperholdripine (**4**, 457 mg, 1.33 mmol, 17%) in the 50% fraction.

### 4.5. Bioassays

Crude methanolic extracts were prepared following standard procedures [7] and assayed against *Saccharomyces cerevisiae* BY4741. S. cerevisiae cells were initially grown on solid YPD medium (2% [*w*/*v*] peptone, 1% [*w*/*v*] yeast extract, 2% [*w*/*v*] glucose) and incubated for 2 days at 30 °C. A single colony was used to inoculate a 10 mL YPD culture, which was incubated at 30 °C for 18 h with shaking. Saturated cultures were diluted 100-fold into liquid YPD, and extracts or purified compounds were diluted into these cultures at indicated concentrations. Samples were arrayed into Honeycomb 100-well plates (Growth Curves USA) and assayed in a Bioscreen C plate reader (Growth Curves USA). OD_600_ readings were taken at 5 min intervals for 18 h with 30 s of shaking before each reading, as previously described. Following the identification of inhibitory targets, the assays were repeated with the purified compounds, initially at a concentration of 100 μM, and then at serial dilutions to calculate the half-maximal inhibitory concentration (IC_50_). Area Under Curve (AUC) values were plotted against the log of the inhibitor concentration, and IC_50_ was calculated in GraphPad Prism using the log(inhibitor) vs. response variable slope model.

## 5. Conclusions

This study validates the ^1^H NMR-based weighted gene co-expression network analysis (WGCNA) approach for identifying bioactive compounds in complex natural product extracts. By isolating and characterizing the compounds responsible for specific bioactivity-associated resonances identified in our previous network analysis, we confirmed that the resonances correlated with antifungal activity indeed correspond to biologically active compounds. This targeted approach led to the discovery of two novel antifungal compounds, including one with a rare imidazolium heterocyclic motif, thereby introducing a new structural class within the *Piper* genus. These findings support the WGCNA method as a reliable tool for natural product discovery, allowing for the efficient identification of ^1^H NMR spectral features linked to bioactivity. This not only streamlines the process of isolating bioactive compounds but also highlights the potential of network-based analyses in guiding the discovery of natural products with therapeutic relevance. Future applications of this approach may extend its utility across other bioactivity assays, further enhancing its role in identifying ecologically relevant and bioactive compounds from crude extracts.

## Figures and Tables

**Table 1:** ^1^H and ^13^C NMR data and assignments for (2’*Z*,6’*E*,10’*E*)-piperoic acid (**1**).

Position	δ_C_, Type ^a^	δ_H_, mult. (*J* in Hz) ^b^	HMBC	NOESY
1	122.2, C			
2	115.1, CH	7.32, d (2.0)	3, 4, 6, 7	
3	145.4, C			
4	149.4, C			
5	137.3, C			
6	124.1, CH	7.35, dt (2.0, 0.5)	2, 4, 7	1’, 2’
7	170.7, C			
1’	28.8, CH_2_	3.33, br d (7.1)	4, 5, 2’, 3’	6, 4’
2’	124.0, CH	5.36, br t (7.4)	1’, 3’, 4’, 13’	6, 13’
3’	129.2, C			
4’	32.9, CH_2_	2.21–2.15, m	5, 2’, 5’, 6’, 13’	1’, 6’, 13’
5’	27.8, CH_2_	2.16–2.09, m	4’, 6’, 7’	14’
6’	125.3, CH	5.16, br t (7.4)	5’, 8’, 14’	4’, 8’
7’	136.2, C			
8’	40.8, CH_2_	1.98–1.92, m	5’, 6’, 7’, 9’, 10’, 14’	6’, 14’
9’	27.8, CH_2_	2.08–2.01, m	6’, 8’, 10’, 11’	14’
10’	125.5, CH	5.07, m	12’, 15’ (weak)	8’, 12’
11’	132.0, C			
12’	25.9, CH_3_	1.65, br s	10’, 11’, 12’	10’
13’	23.8, CH_3_	1.75, br q (1.2)	5, 2’, 3’, 4’	2’, 4’
14’	16.1, CH_3_	1.60, br s	6’, 7’, 8’	5’
15’	17.7, CH_3_	1.58, br s	10’, 11’, 15’	

^a 13^C NMR recorded at 101 MHz in CD_3_OD; ^b 1^H NMR recorded at 400 MHz in CD_3_OD.

**Table 2:** ^1^H and ^13^C NMR assignments for macatrichocarpin B (**2**).

Position	δ_C_, Type ^a^	δ_H_, mult. (*J* in Hz) ^b^	HMBC
1	105.3, C		
2/6	165.2, C		
3/5	95.9, CH	5.86, s	1, 2/6, 4, 7
4	164.9, C		
7	205.9, C		
8	46.7, CH_2_	3.27–3.24, m	7, 9, 10
9	30.5, CH_2_	2.85, dd (8.2, 6.7)	7, 8, 10, 11, 15
10	134.7, C		
11	130.5, CH	6.99, d (2.0)	9, 13, 15, 1’
12	130.6, C		
13	156.6, C		
14	111.6, CH	6.79, d (8.3)	10, 11, 12, 13, 15
15	127.7, CH	7.00, dd (8.2, 2.3)	11, 13, 14
16	56.1, CH_3_	3.78, s	
1’	29.3, CH_2_	3.25, br d (7.4)	11, 12, 13, 2’, 3’
2’	123.7, CH	5.25, m	5’ (weak)
3’	133.0, C		
4’	25.9, CH_3_	1.71, br s	2’, 3’, 5’
5’	17.8, CH_3_	1.69, br s	2’, 3’, 4’

^a 13^C NMR recorded at 101 MHz in CD_3_OD; ^b 1^H NMR recorded at 400 MHz in CD_3_OD.

**Table 3:** ^1^H and ^13^C NMR assignments for 14-prenylmacatrichocarpin B (**3**).

Position	δ_C_, Type ^a^	δ_H_, mult. (*J* in Hz) ^b^	HMBC
1	105.3, C		
2/6	165.1, C		
3/5	95.8, CH	5.86, s	1, 2/6, 4, 7
4	164.7, C		
7	206.0, C		
8	46.8, CH_2_	3.21–3.27, m	7, 9, 10
9	30.6, CH_2_	2.79, t (7.6)	7, 8, 10, 11/15
10	134.4, C		
11/15	128.2, CH	6.79, s	9, 13, 1’/1’’
12/14	128.8, C		
13	151.1, C		
1’/1’’	29.7, CH_2_	3.25, br d (7.3)	11/15, 13, 12/14, 2’/2’’, 3’/3’’, 5’/5’’
2’/2’’	123.4, CH	5.26, m	1’/1’’, 4’/4’’, 5’/5’’
3’/3’’	133.7, C		
4’/4’’	25.8, CH_3_	1.72, br s	2’/2’’, 3’/3’’, 5’/5’’
5’/5’’	17.8, CH_3_	1.71, br s	2’/2’’, 3’/3’’, 4’/4’’

^a 13^C NMR recorded at 101 MHz in CD_3_OD. ^b 1^H NMR recorded at 400 MHz in CD_3_OD.

**Table 4:** ^1^H and ^13^C NMR assignments for piperholdripine (**4**).

Position	δ_C_, Type ^a^	δ_H_, mult. (*J* in Hz) ^b^	HMBC
1	31.6, CH_2_	3.47, br t (7.8)	8, 9
2	22.1, CH_2_	2.50, br pent. (7.8)	1,3,9
3	60.5, CH_2_	4.83, brt (7.8)	1,2,5,9
4	N		
5	138.8, CH	8.60, br s	6,7,9
6	142.9, C		
7	145.9, C	8.17, br s	5,9
8	140.7, C		
9	156.6, C		
1’/1’’	33.0, CH_2_	2.80, br t (7.3)	2’/2’’, 5, 6, 7, 8, 9
2’/2’’	31.7, CH_2_	1.66–1.72, m	8, 6, 1’/1’’, 3’/3’’-7’/7’’
3’/3’’-4’/4’’	30.2, 30.1, CH_2_	1.34–1.44, m	
5’/5’’-7’,7’’	32.7, 33.2, 23.7, CH_2_	1.26–1.36, m	
8’/8’’	14.4 CH_3_	0.90, m	

^a 13^C NMR recorded at 125 MHz in CD_3_OD; ^b 1^H NMR recorded at 500 MHz in CD_3_OD.

**Table 5:** Growth-inhibition activity of crude extracts and isolated compounds against *S. cerevisiae*.

Species and Isolated Compound(s)	% Inhibition	IC50 (μM)
*P. pseudobumbratum*	63.2	
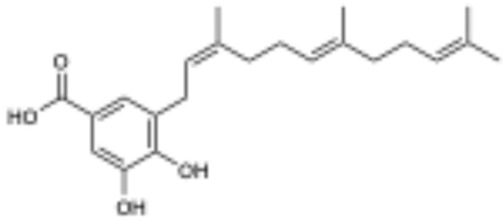 (2’*Z*,6’*E*,10’*E*)-piperoic acid (**1**) ***active***	28.5	Not Determined
*P. peracuminatum*	98.7	
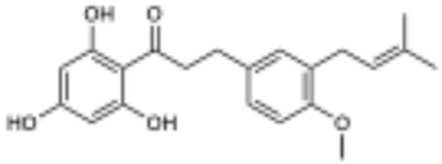 macatrichocarpin B (**2**) ***active***	75.3	10.5 µM
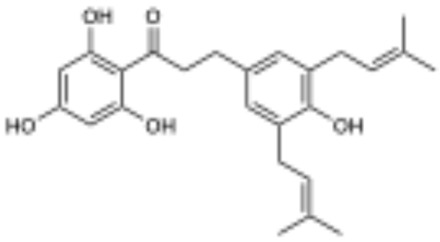 5-prenyl macatrichocarpin B (**3**) ***not active***	0.0	>100 µM
*P. holdrigeanum*	93.8	
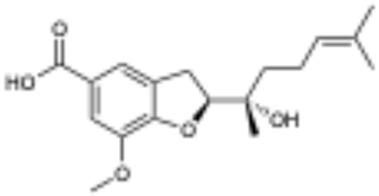 **5**	0.0	>100 μM
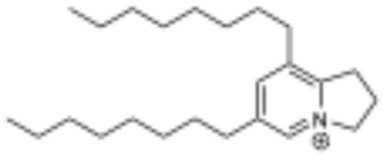 piperholdripine (**4**)	85.3	4.9 µM

## Data Availability

The raw NMR data for compounds **1**–**5** are deposited into the Natural Products Magnetic Resonance Database (NP-MRD; www.np-mrd.org), accessed on 10 May 2025.

## Supplementary Materials

The following supporting information can be downloaded at: https://www.mdpi.com/article/10.3390/molecules30092020/s1, Figure S1: Growth-inhibition activity of *Piper* extracts against *S. cerevisiae*; Figure S2: ^1^H NMR Spectrum (400 MHz) of 1 in CD_3_OD; Figure S3: ^13^C NMR Spectrum (101 MHz) of 1 in CD_3_OD; Figure S4: ^1^H-^1^H COSY NMR Spectrum (400 MHz) of 1 in CD_3_OD; Figure S5: HSQC NMR Spectrum (400/101 MHz) of 1 in CD_3_OD; Figure S6: HMBC NMR Spectrum (400/101 MHz) of 1 in CD_3_OD; Figure S7: ^1^H-^1^H NOESY NMR Spectrum (400 MHz) of 1 in CD_3_OD; Figure S8: ^1^H NMR Spectrum (400 MHz) of 2 in CD_3_OD; Figure S9: ^1^H NMR Spectrum (400 MHz) of 2 in CD_3_CN; Figure S10: ^13^C NMR Spectrum (101 MHz) of 2 in CD_3_CN; Figure S11: ^1^H-^1^H COSY NMR Spectrum (400 MHz) of 2 in CD_3_CN; Figure S12: HSQC NMR Spectrum (400/101 MHz) of 2 in CD_3_CN; Figure S13: HMBC NMR Spectrum (400/101 MHz) of 2 in CD_3_CN; Figure S14: ^1^H NMR Spectrum (400 MHz) of 3 in CD_3_CN; Figure S15: ^13^C NMR Spectrum (101 MHz) of 3 in CD_3_CN; Figure S16: ^1^H-^1^H COSY NMR Spectrum (400 MHz) of 3 in CD_3_CN; Figure S17: HSQC NMR Spectrum (400/101 MHz) of 3 in CD_3_CN; Figure S18: HMBC NMR Spectrum (400/101 MHz) of 3 in CD_3_CN; Figure S19: ^1^H NMR Spectrum (400 MHz) of 4 in CD_3_OD; Figure S20: Expanded ^1^H NMR Spectrum (400 MHz) of 4 in CD_3_OD, highlighting peaks in the 0–3.5 ppm region; Figure S21: ^13^C NMR Spectrum (101 MHz) of 4 in CD_3_OD; Figure S22: ^1^H-^1^H COSY NMR Spectrum (400 MHz) of 4 in CD_3_OD; Figure S23: HSQC NMR Spectrum (400/101 MHz) of 4 in CD_3_OD; Figure S24: HMBC NMR Spectrum (400/101 MHz) of 4 in CD_3_OD; Figure S25: ^1^H NMR Spectrum (400 MHz) of synthesized 4 in CD_3_OD; Figure S26: ^13^C NMR Spectrum (101 MHz) of synthesized 4 in CD_3_OD; Figure S27: ^1^H-^1^H COSY NMR Spectrum (400 MHz) of synthesized 4 in CD_3_OD; Figure S28: HSQC NMR Spectrum (400/101 MHz) of synthesized 4 in CD_3_OD; Figure S29: HMBC NMR Spectrum (400/101 MHz) of synthesized 4 in CD_3_OD; Figure S30: ^1^H NMR Spectrum (400 MHz) of 5 in CDCl_3_; Figure S31: ^13^C NMR Spectrum (101 MHz) of 5 in CDCl_3_.
