# Supplementary material for: 1H-NMR Guided Isolation of Bioactive Compounds from Species of the Genus Piper
Source: Molecules. 2025 Apr 30;30(9):2020. doi: 10.3390/molecules30092020 (PMC12073215; doi:10.3390/molecules30092020)

Supporting Information

^{1}H-NMR Resonance Guided Isolation of Bioactive Compounds from Species of the Genus Piper

Celso R. Oliveira,[2] Megan J. Burroughs,[1] Lora A. Richards,[3] Lee A. Dyer,[3] Federico Urbano-Muñoz,[4] Camryn Lee,[4] Megan Warner,[4] Craig D. Dodson,[1] Ian S. Wallace,[4,5*] Christopher S. Jeffrey[1]

[1]Hitchcock Center for Chemical-Ecology and Department of Chemistry, University of Nevada—Reno, Reno, NV 89557-0216

[2]University of Wisconsin—Madison, Department of Forestry and Wildlife Ecology, Madison, WI 53706

[3]Hitchcock Center for Chemical-Ecology and Department of Biology, University of Nevada—Reno, Reno, NV 89557-0216

[4]Department of Biochemistry and Molecular Biology, University of Nevada, Reno- Reno, NV 89557

[5]Complex Carbohydrate Research Center, University of Georgia, Athens, GA 30606

*Christopher S. Jeffrey, cjeffrey@unr.edu and Ian S. Wallace Ian.Wallace@uga.edu

Table of Contents

Growth Inhibition Studies

Figure S1. Growth-inhibition activity of Piper extracts against S. cereviseae 4

Characterization Data for Compound 1

Figure S2. ^{1}H NMR Spectrum (400 MHz) of 1 in CD_3OD. 5

Figure S3. ^{13}C NMR Spectrum (101 MHz) of 1 in CD_3OD. 6

Figure S4. ^{1}H-^{1}H COSY NMR Spectrum (400 MHz) of 1 in CD_3OD. 7

Figure S5. HSQC NMR Spectrum (400/101 MHz) of 1 in CD_3OD. 8

Figure S6. HMBC NMR Spectrum (400/101 MHz) of 1 in CD_3OD. 9

Figure S7. ^{1}H-^{1}H NOESY NMR Spectrum (400 MHz) of 1 in CD_3OD. 10

Characterization Data for Compound 2

Figure S8. ^{1}H NMR Spectrum (400 MHz) of 2 in CD_3OD. 11

Figure S9. ^{1}H NMR Spectrum (400 MHz) of 2 in CD_3CN. 12

Figure S10. ^{13}C NMR Spectrum (101 MHz) of 2 in CD_3CN. 13

Figure S11. ^{1}H-^{1}H COSY NMR Spectrum (400 MHz) of 2 in CD_3CN. 14

Figure S12. HSQC NMR Spectrum (400/101 MHz) of 2 in CD_3CN. 15

Figure S13. HMBC NMR Spectrum (400/101 MHz) of 2 in CD_3CN. 16

Characterization Data for Compound 3

Figure S14. ^{1}H NMR Spectrum (400 MHz) of 3 in CD_3CN. 17

Figure S15. ^{13}C NMR Spectrum (101 MHz) of 3 in CD_3CN. 18

Figure S16. ^{1}H-^{1}H COSY NMR Spectrum (400 MHz) of 3 in CD_3CN. 19

Figure S17. HSQC NMR Spectrum (400/101 MHz) of 3 in CD_3CN. 20

Figure S18. HMBC NMR Spectrum (400/101 MHz) of 3 in CD_3CN. 21

Characterization Data for Compound 4

Figure S19. ^{1}H NMR Spectrum (400 MHz) of 4 in CD_3OD. 22

Figure S20. Expanded ^{1}H NMR Spectrum (400 MHz) of 4 in CD_3OD, highlighting the peaks in the 0–3.5 ppm region. 23

Figure S21. ^{13}C NMR Spectrum (101 MHz) of 4 in CD_3OD 24

Figure S22. ^{1}H-^{1}H COSY NMR Spectrum (400 MHz) of 4 in CD_3OD. 25

Figure S23. HSQC NMR Spectrum (400/101 MHz) of 4 in CD_3OD. 26

Figure S24. HMBC NMR Spectrum (400/101 MHz) of 4 in CD_3OD. 27
Characterization Data for synthesized Compound 4
Figure S25. 1H NMR Spectrum (400 MHz) of Synthesized 4 in CD3OD. 28
Figure S26. ^{13}C NMR Spectrum (101 MHz) of synthesized 4 in CD_3OD.............................29
Figure S27. ^{1}H-^{1}H COSY NMR Spectrum (400 MHz) of synthesized 4 in CD_3OD. 30
Figure S28. HSQC NMR Spectrum (400/101 MHz) of synthesized 4 in CD_3OD................31
Figure S29. HMBC NMR Spectrum (400/101 MHz) of synthesized 4 in CD_3OD...............32
Characterization Data for Compound 5
Figure S30 ^{1}H NMR Spectrum (400 MHz) of 5 in $CDCl_3$..33
Figure S31. ^{13}C NMR Spectrum (101 MHz) of 5 in $CDCl_3$. 34

Figure S1. Growth-inhibition activity of Piper extracts against S. cereviseae. Relative yeast growth was calculated as the ratio between the area under curve (AUC) of extract assays and the control (YPD + yeast + methanol).

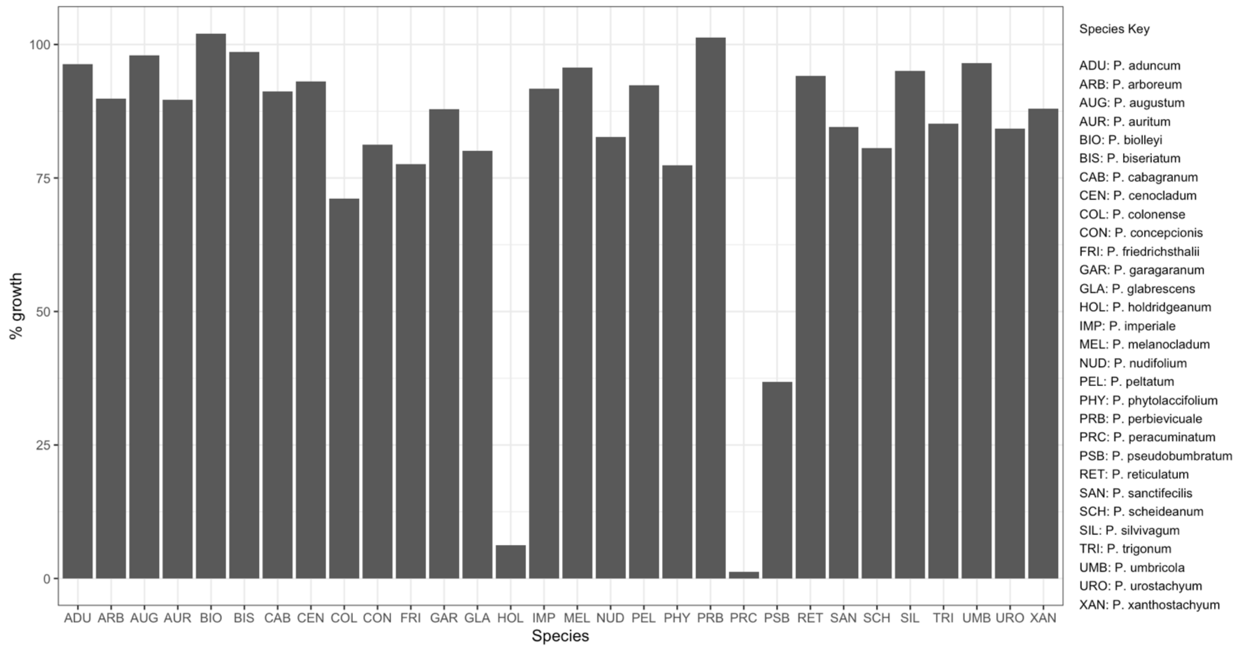

Figure S2. ^{1}H NMR Spectrum (400 MHz) of **1** in CD_3OD.

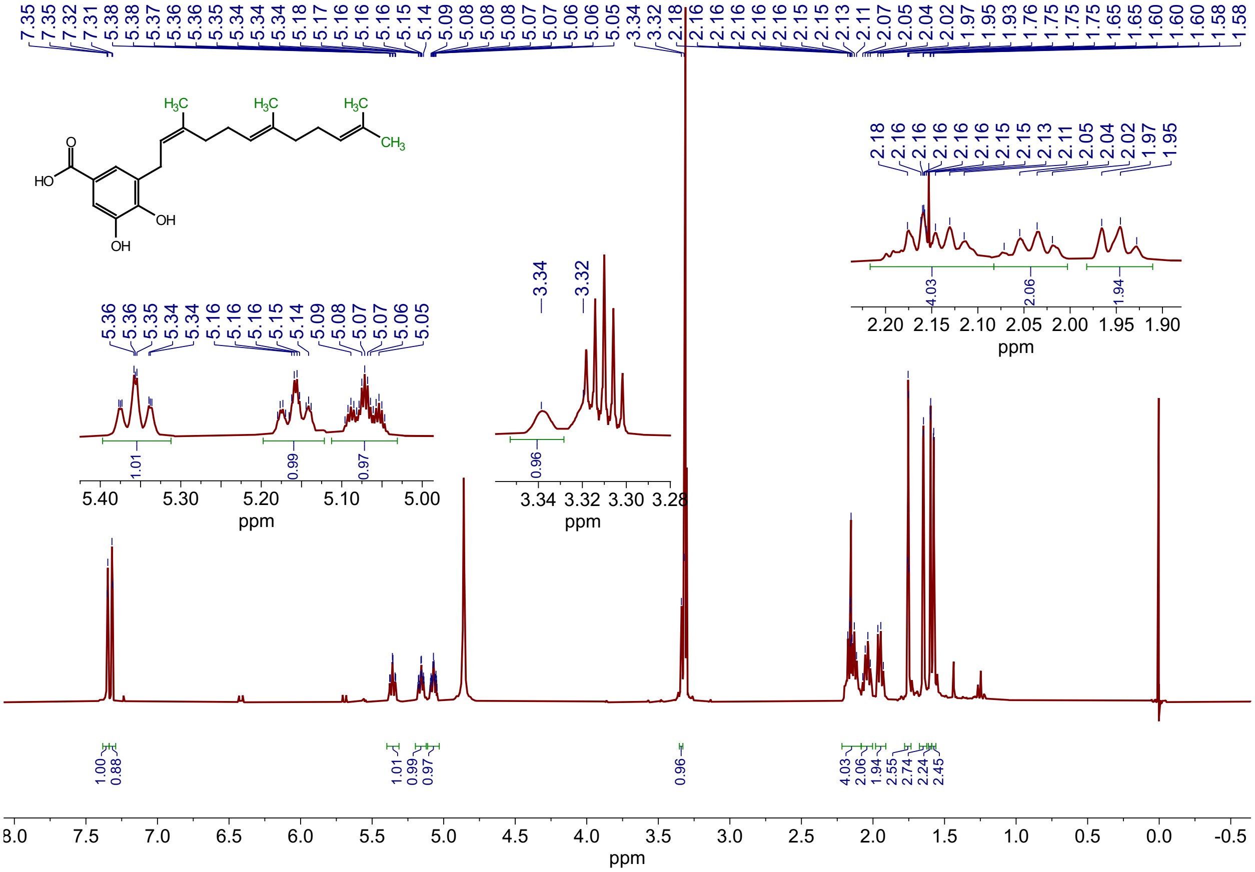

Figure S3. ^{13}C NMR Spectrum (101 MHz) of **1** in CD_3OD.

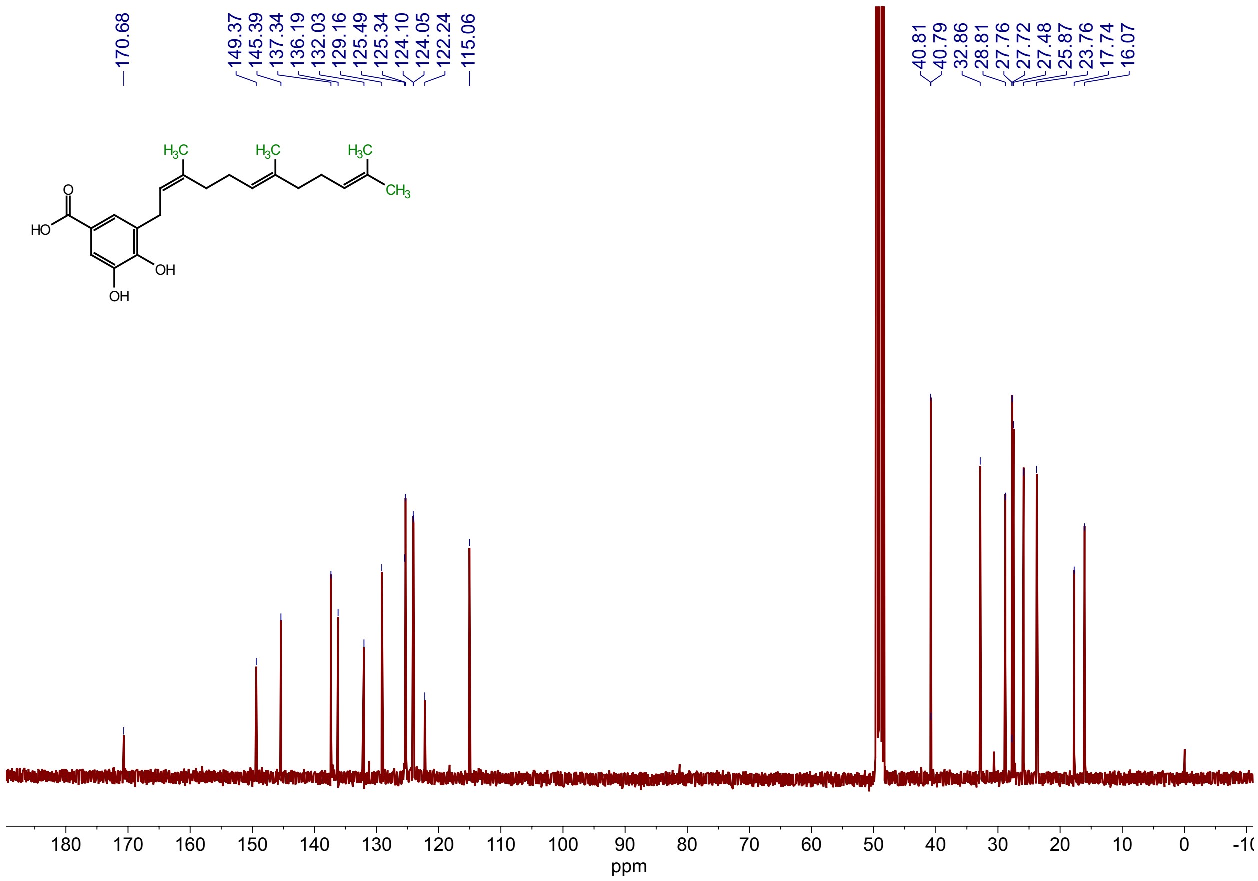

Figure S4. ^{1}H-^{1}H COSY NMR Spectrum (400 MHz) of **1** in CD_3OD.

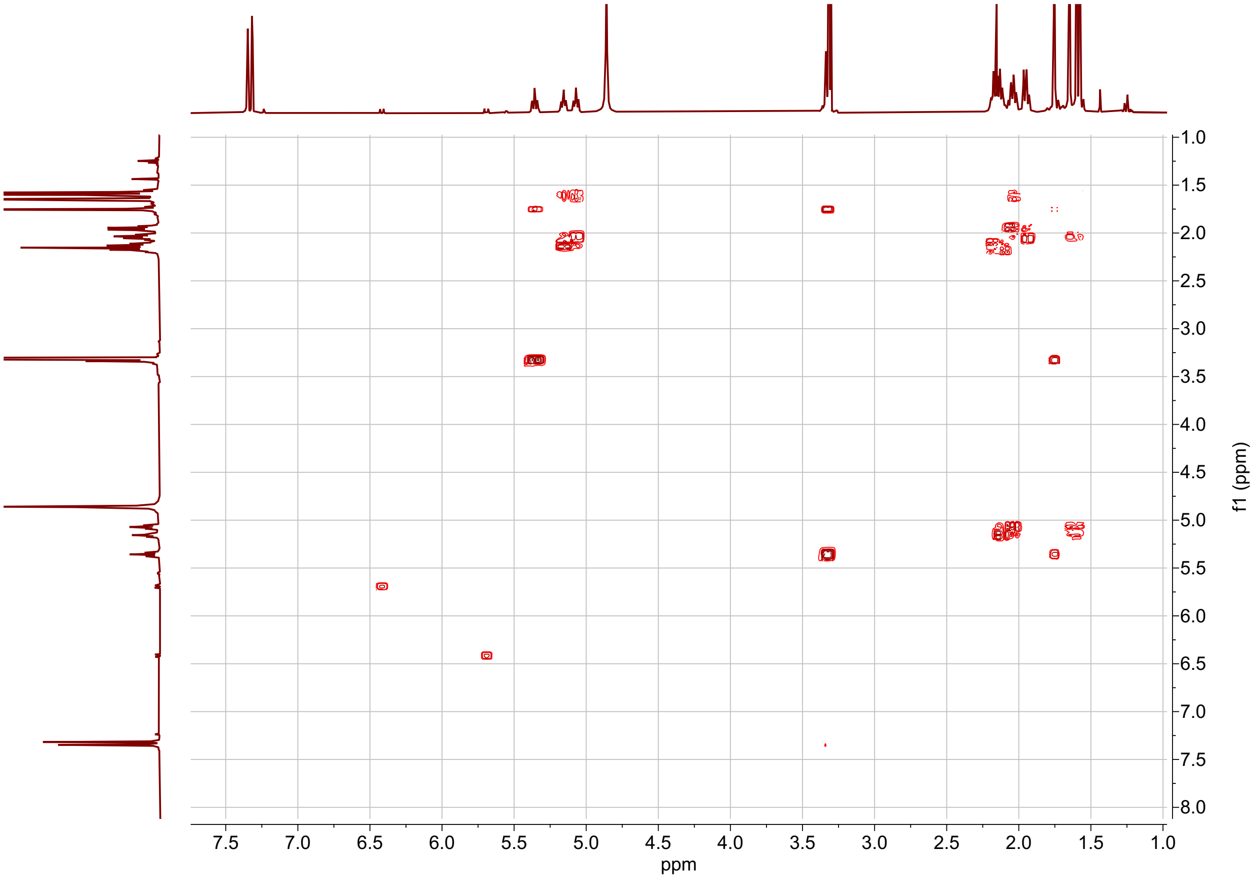

Figure S5. HSQC NMR Spectrum (400/101 MHz) of **1** in CD_3OD.

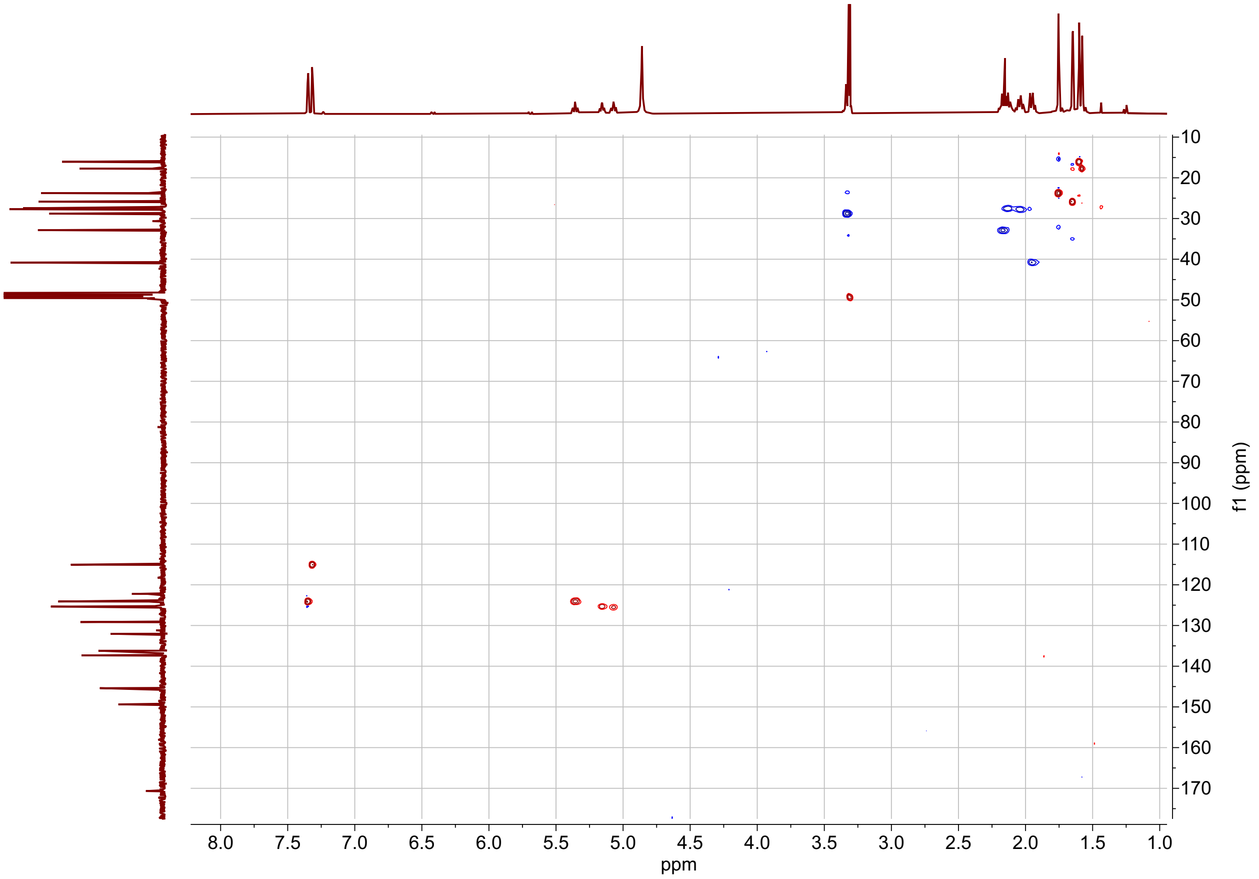

Figure S6. HMBC NMR Spectrum (400/101 MHz) of **1** in CD_3OD.

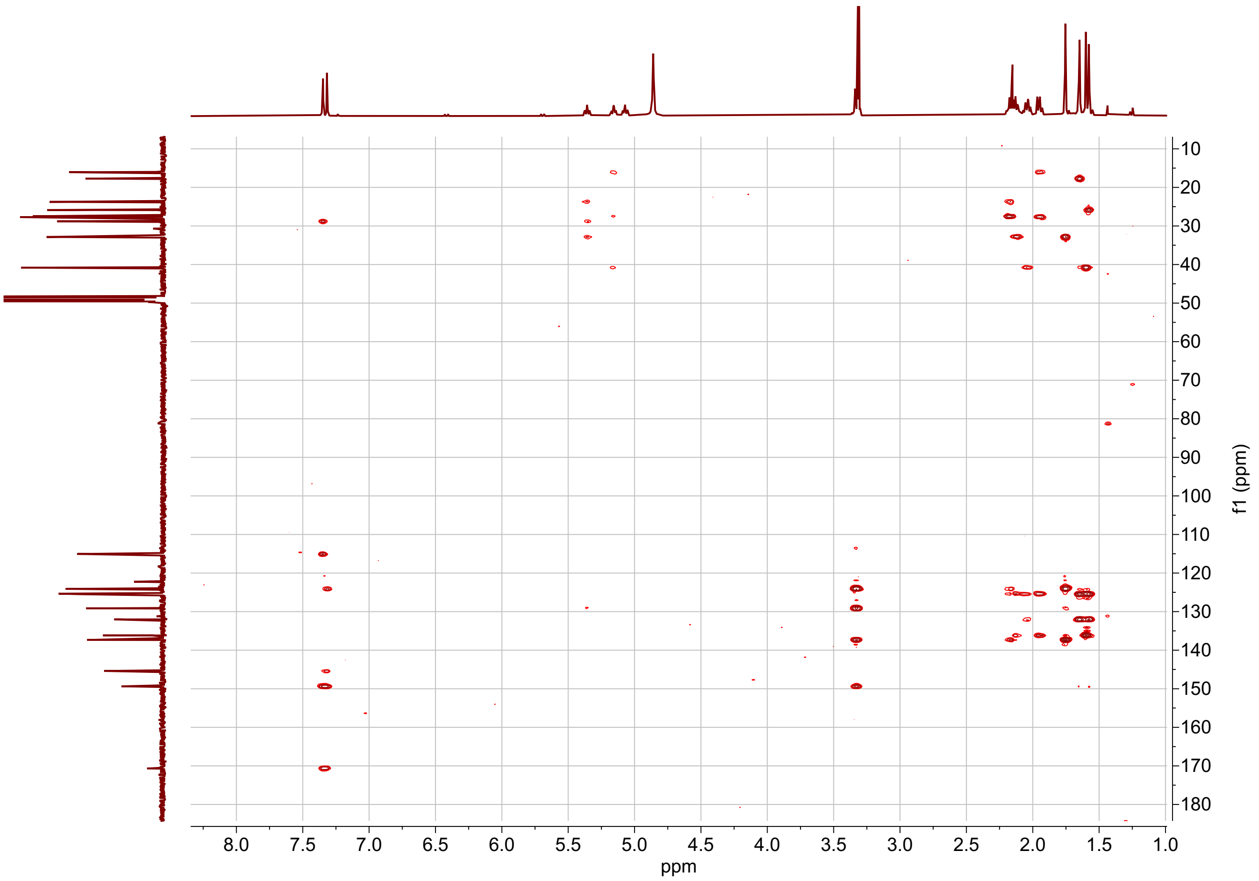

Figure S7. ^{1}H-^{1}H NOESY NMR Spectrum (400 MHz) of **1** in CD_3OD.

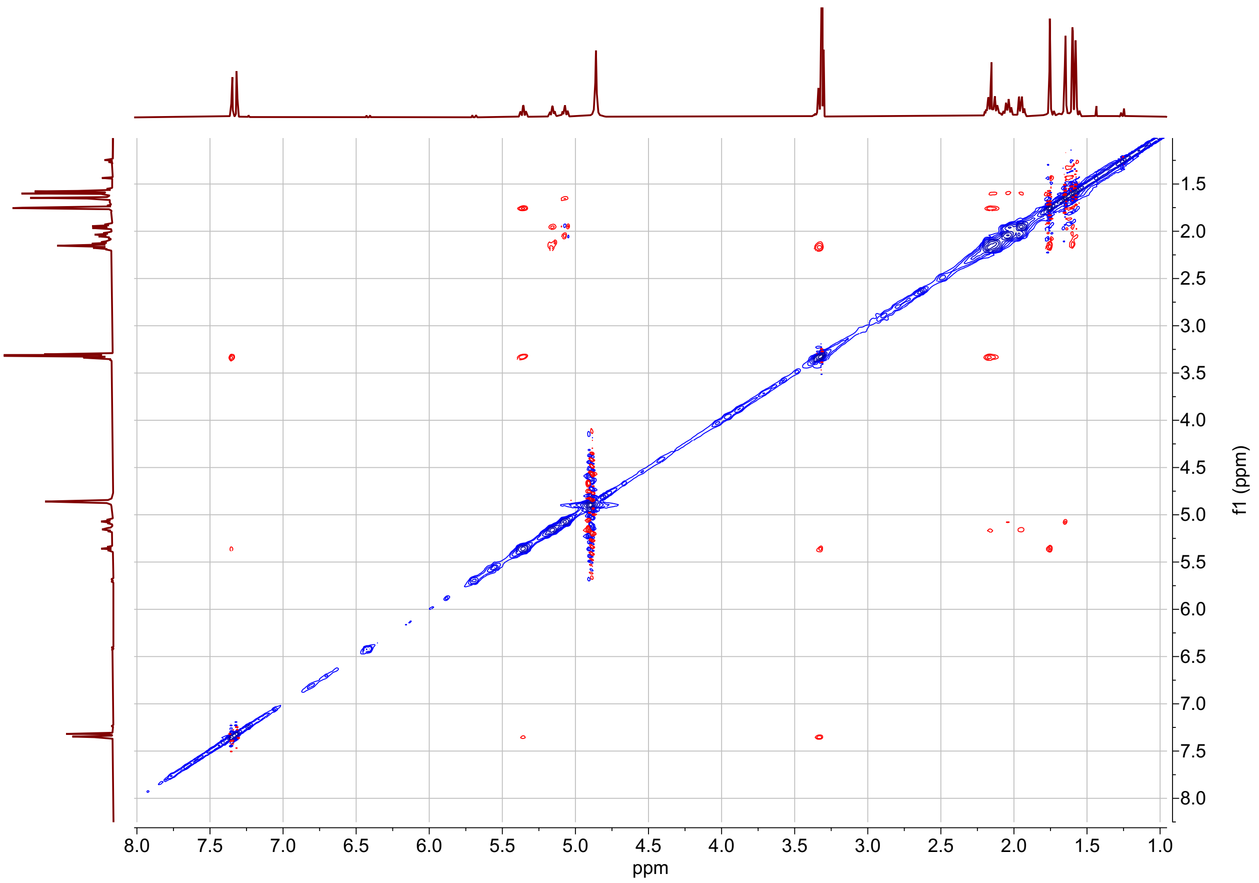

Figure S8. ^{1}H NMR Spectrum (400 MHz) of **2** in CD_3OD.

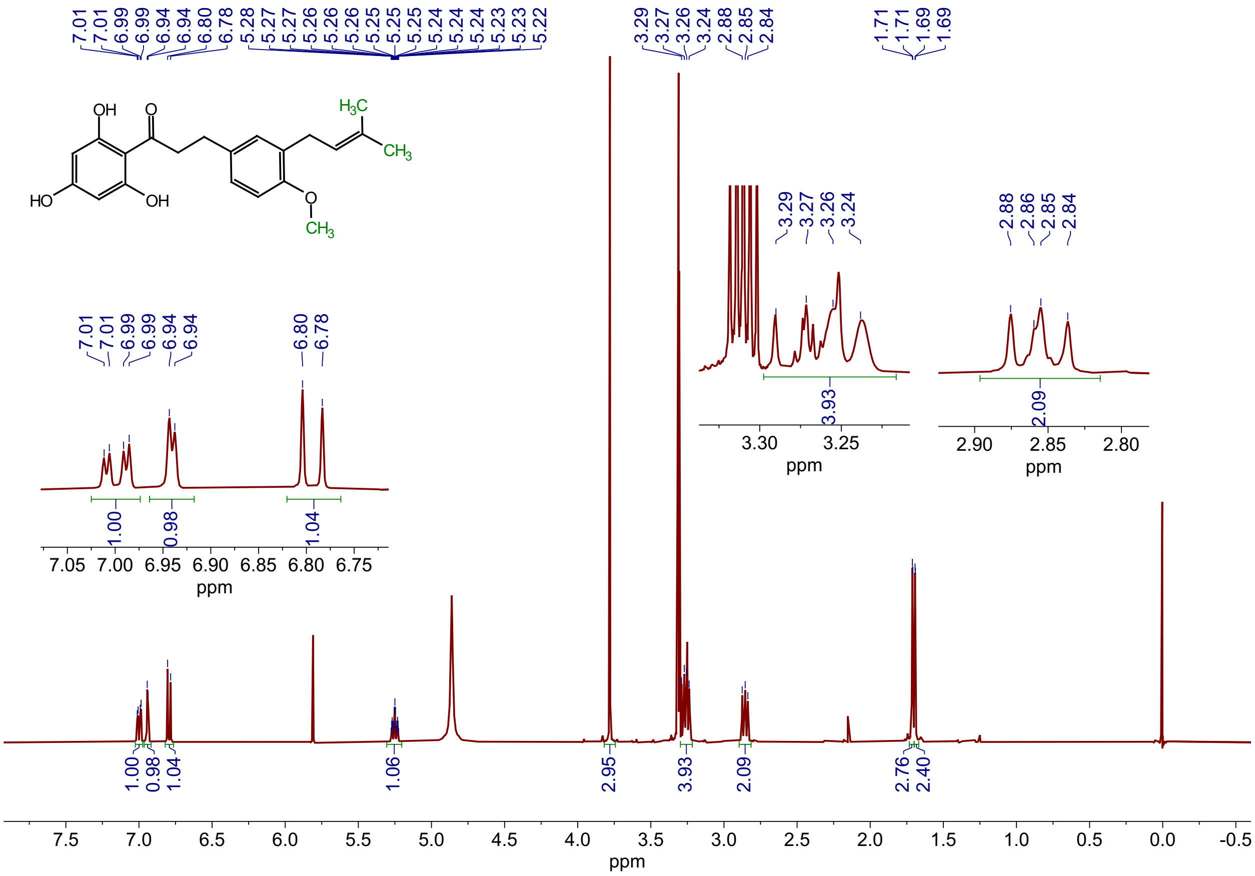

Figure S9 ^{1}H NMR Spectrum (400 MHz) of **2** in CD_3CN.

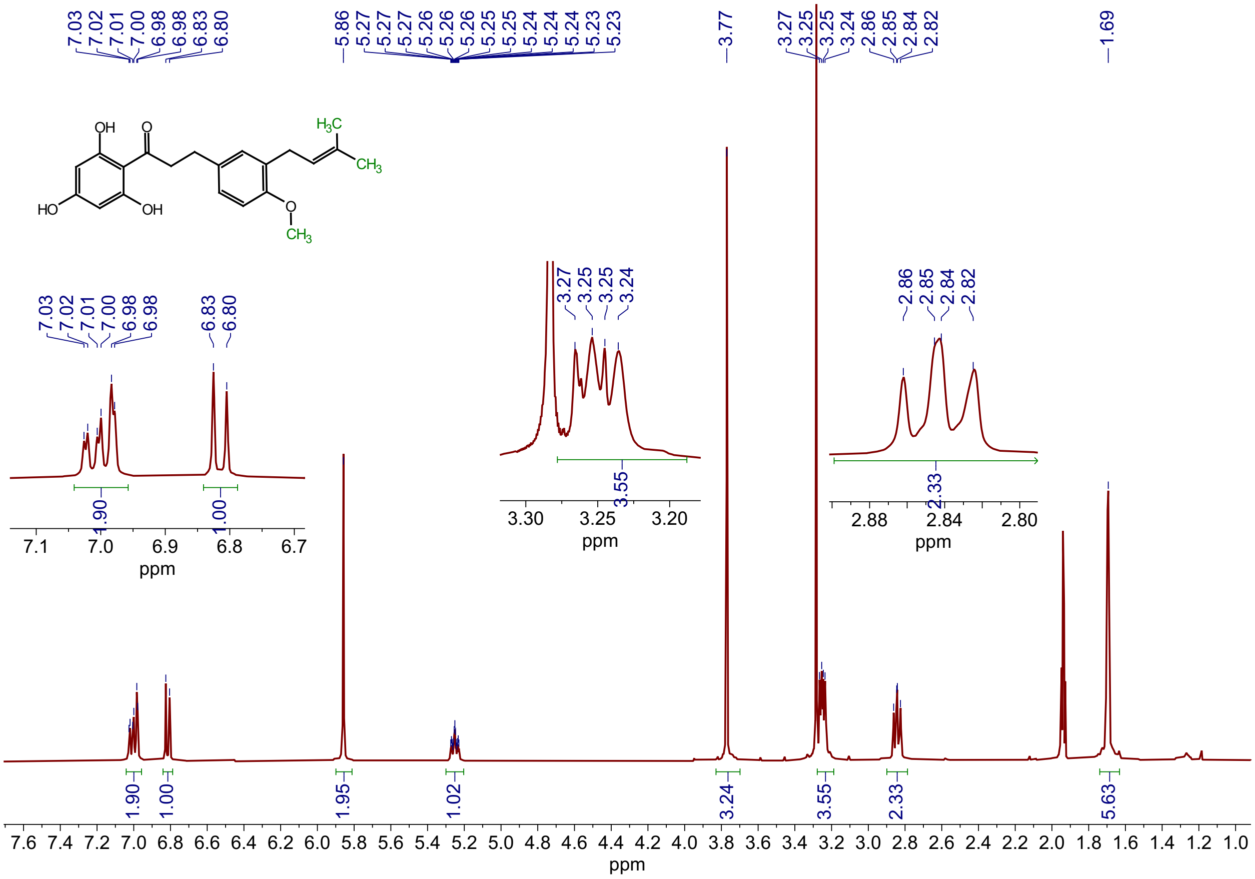

Figure S10. ^{13}C NMR Spectrum (101 MHz) of **2** in CD_3CN.

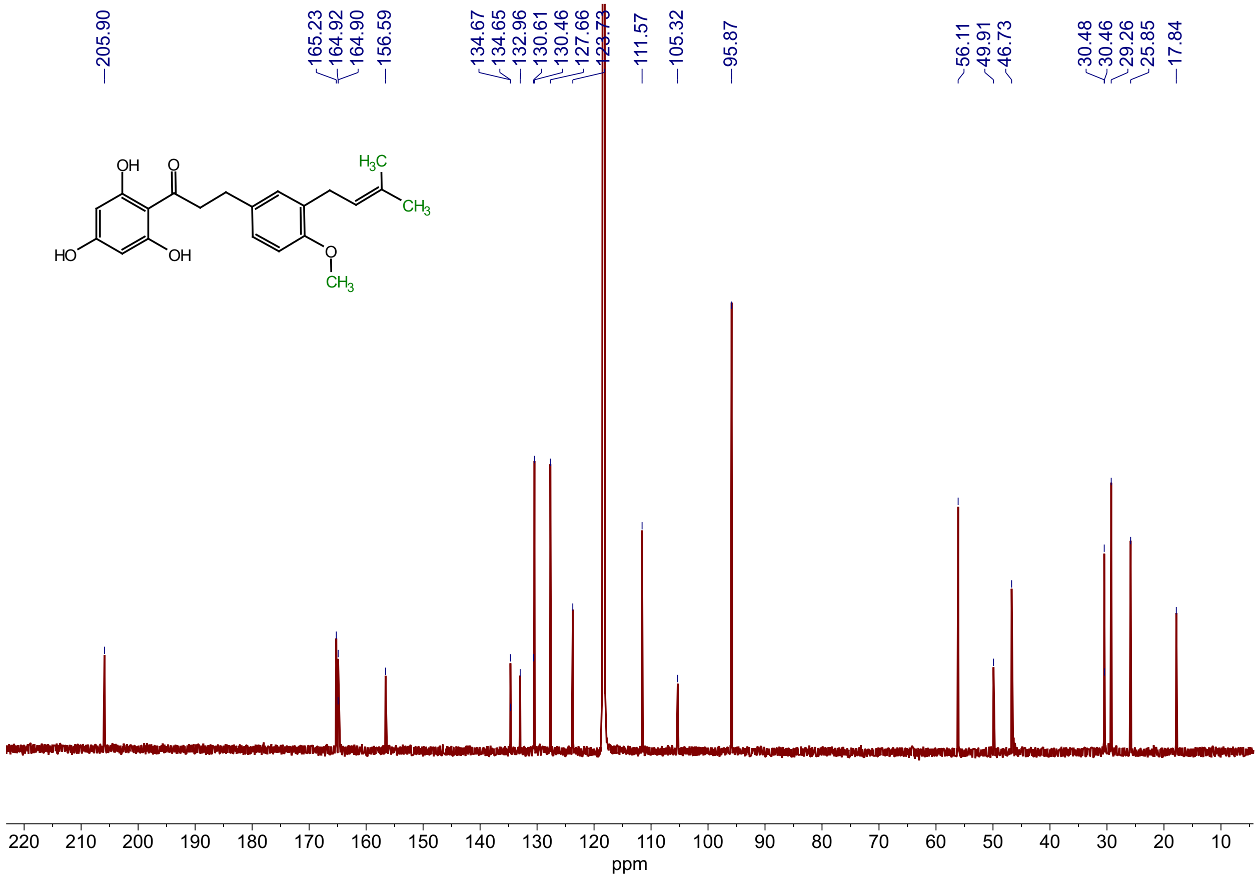

Figure S11. ^1H-^1H COSY NMR Spectrum (400 MHz) of **2** in CD_3CN.

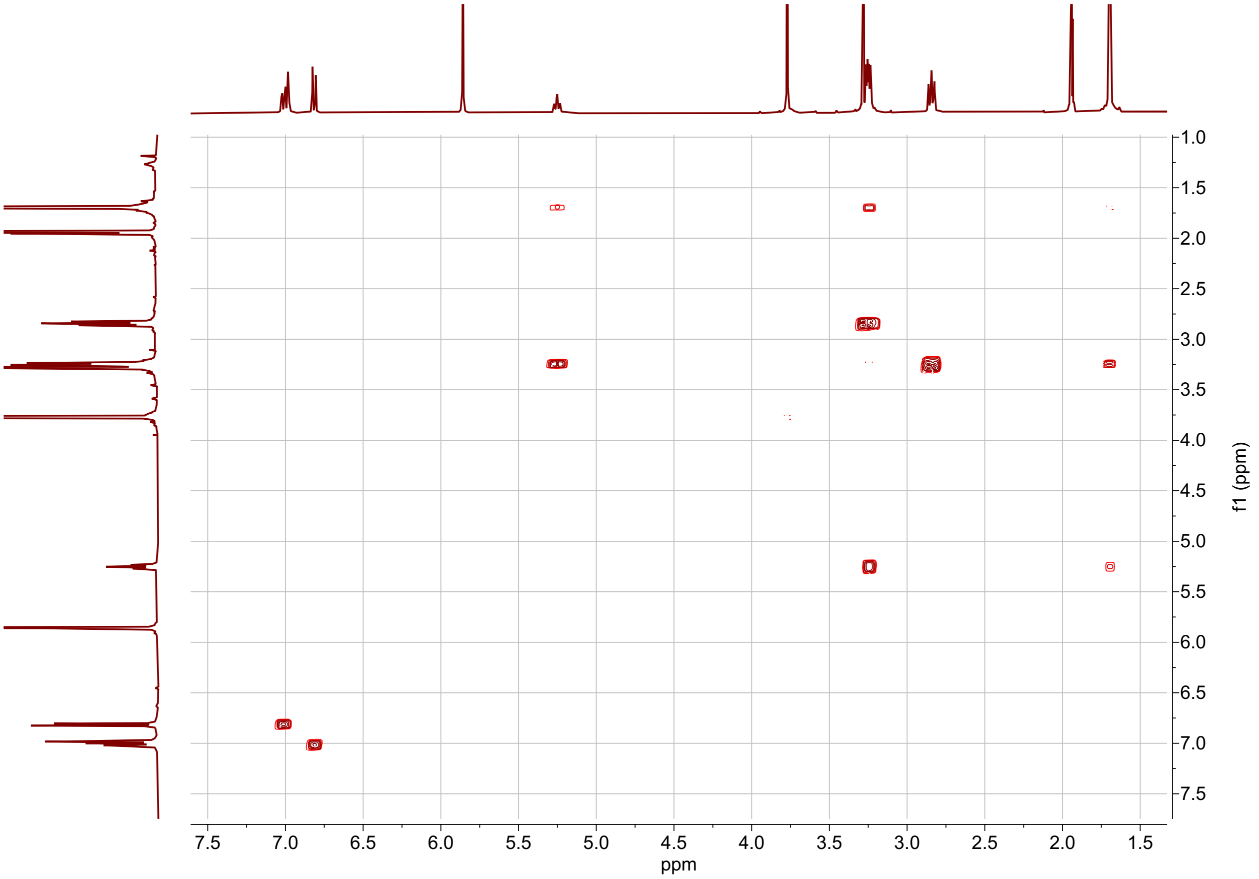

Figure S12. HSQC NMR Spectrum (400/101 MHz) of **2** in CD_3CN.

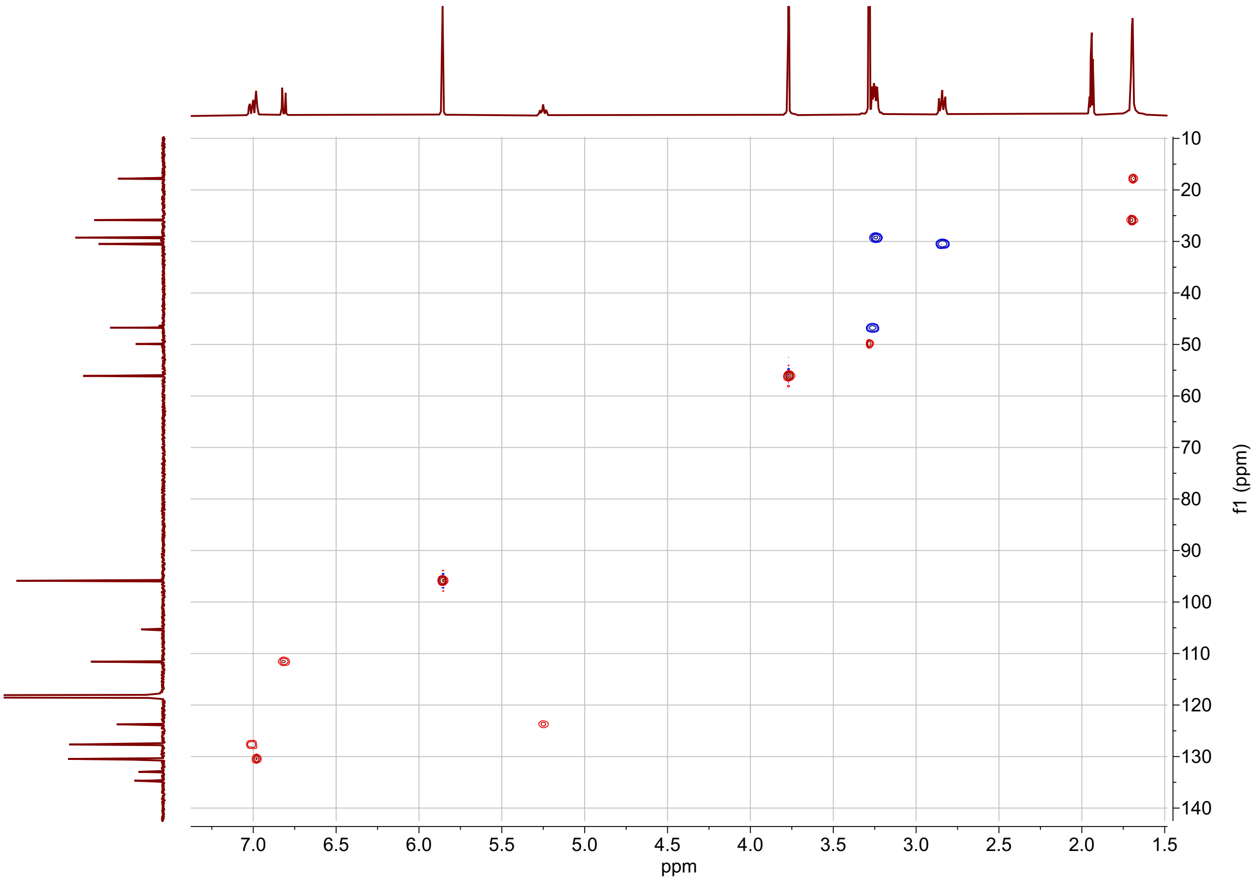

Figure S13. HMBC NMR Spectrum (400/101 MHz) of **2** in CD_3CN.

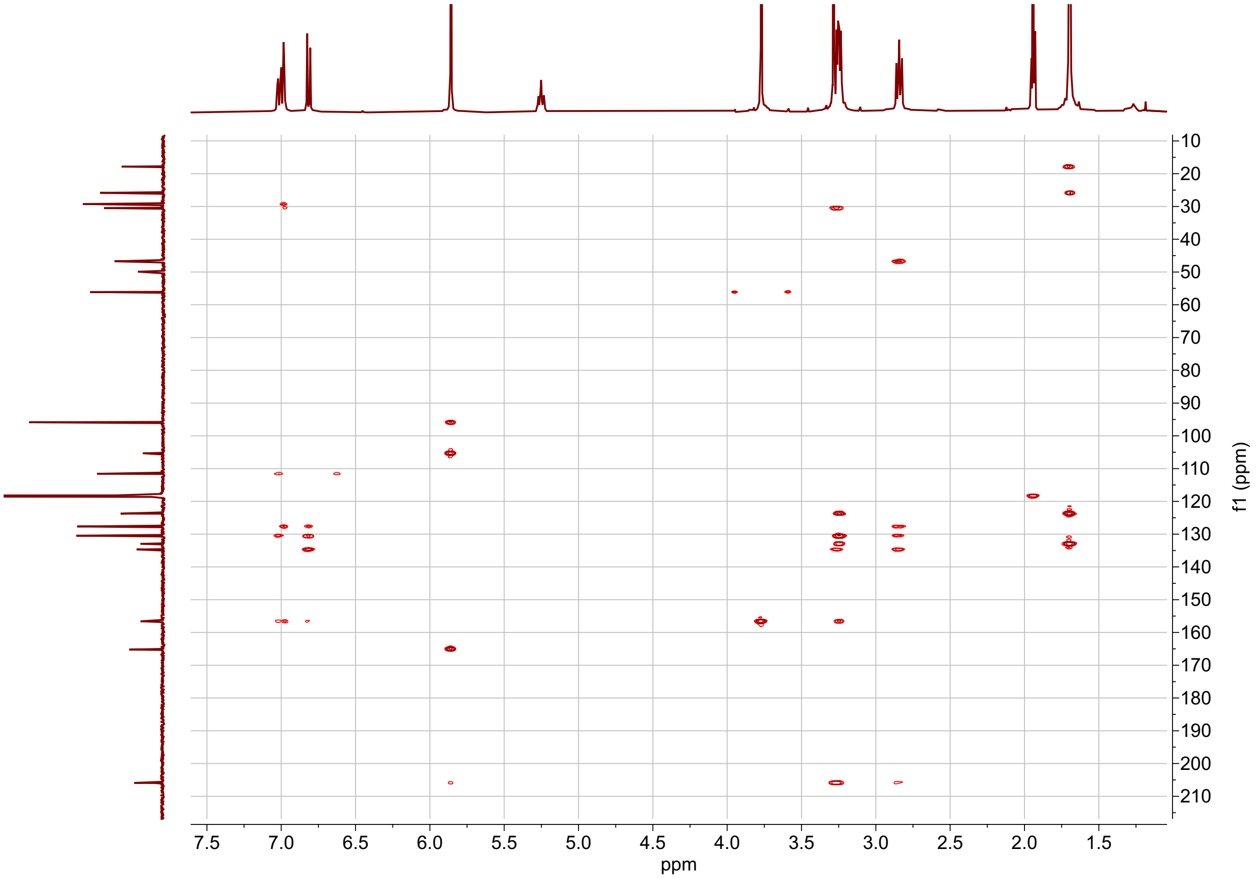

Figure S14. ^{1}H NMR Spectrum (400 MHz) of **3** in CD_3CN.

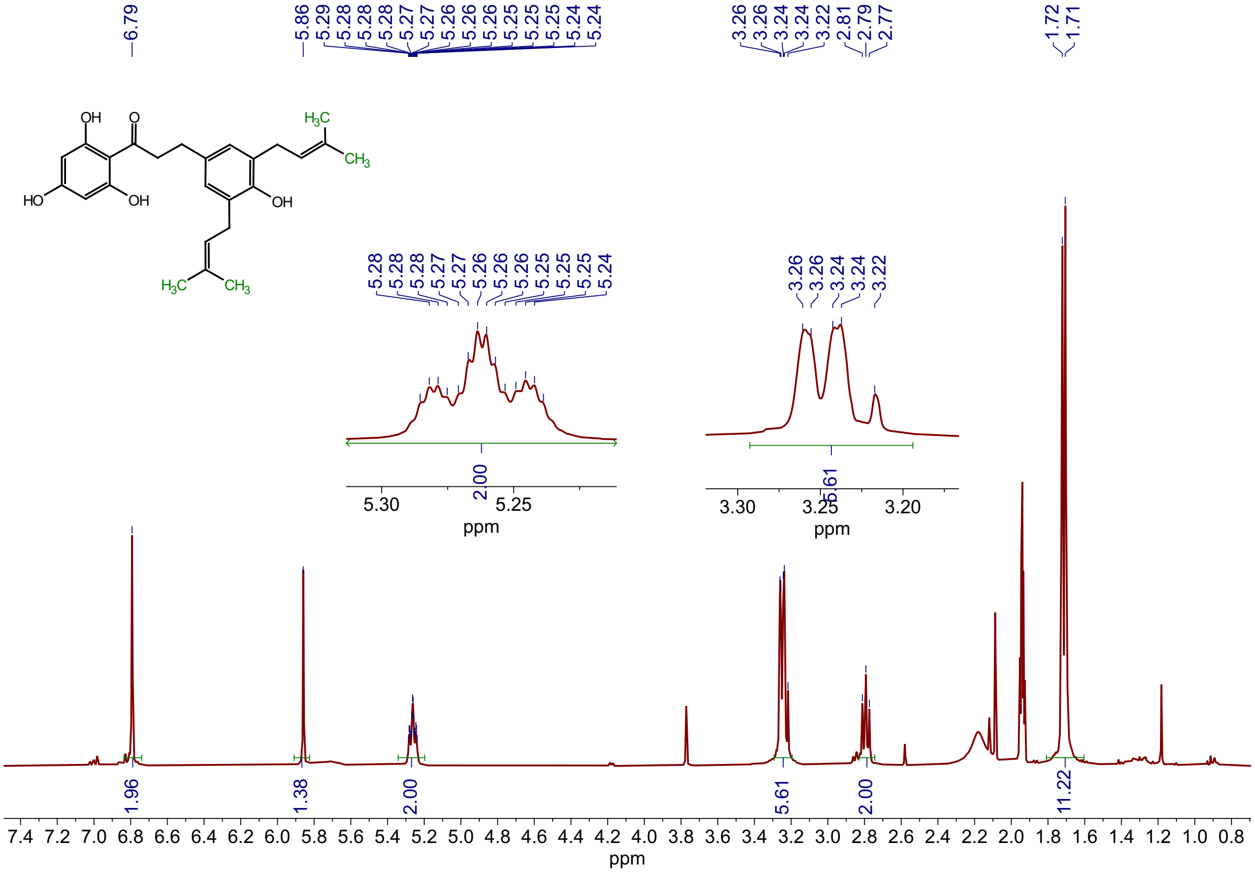

Figure S15. ^{13}C NMR Spectrum (101 MHz) of **3** in CD_3CN.

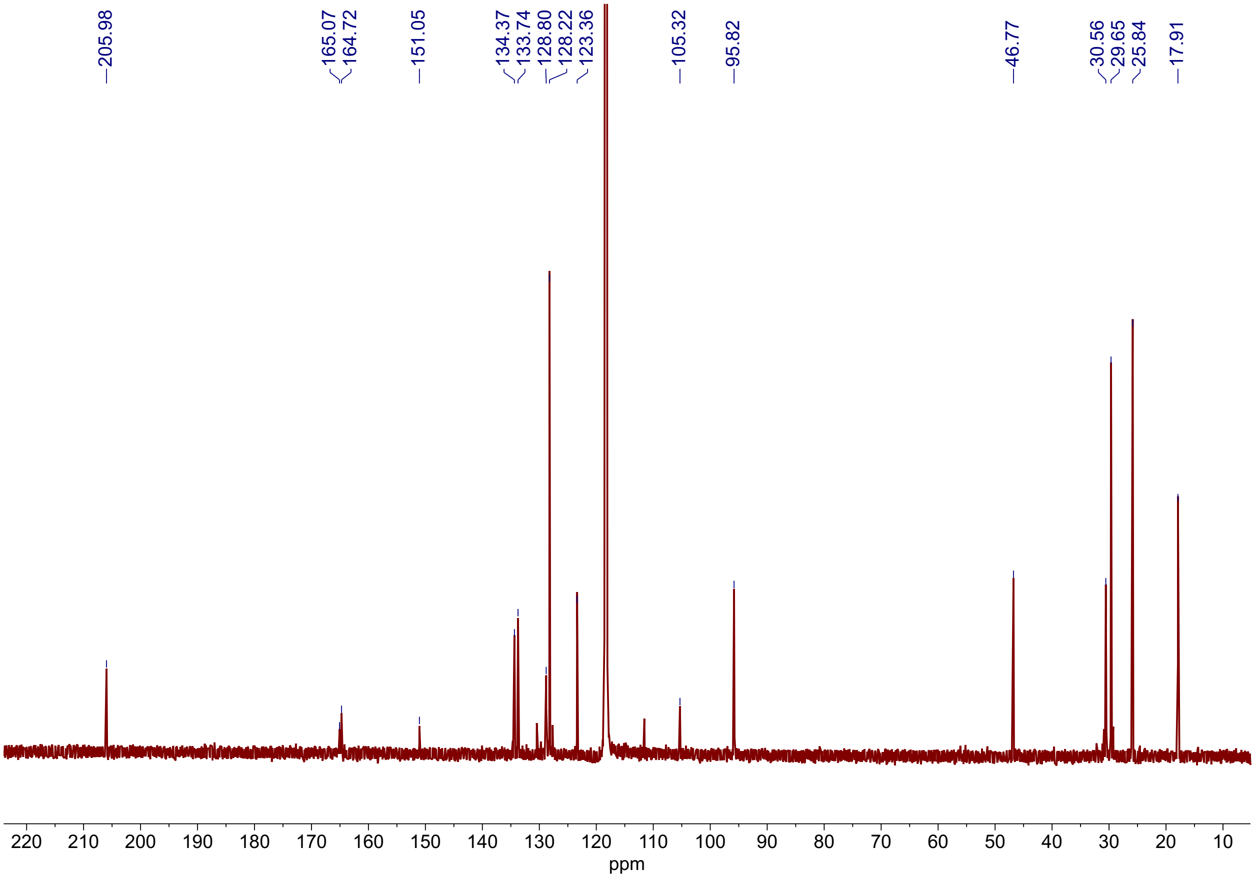

Figure S16. ^1H-^1H COSY NMR Spectrum (400 MHz) of **3** in CD_3CN.

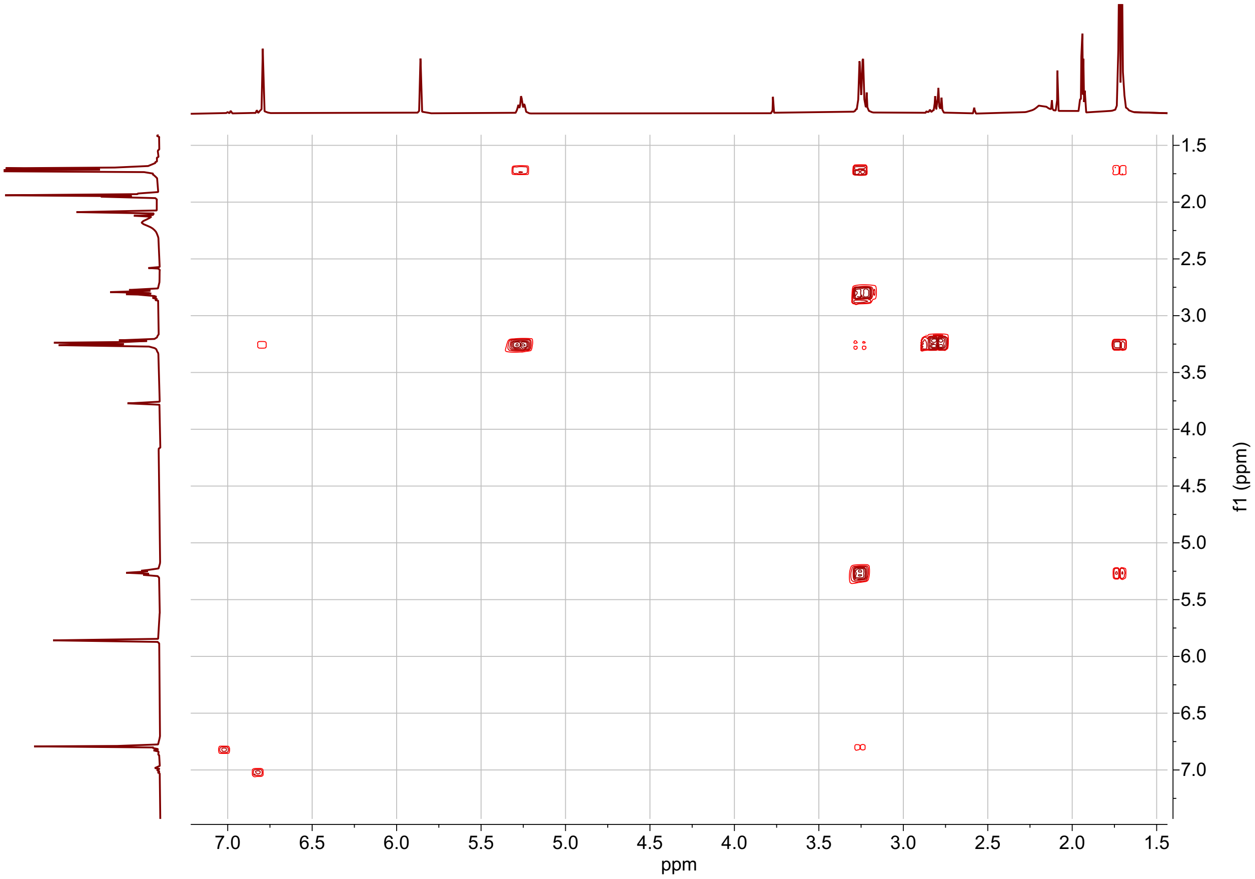

Figure S17. HSQC NMR Spectrum (400/101 MHz) of **3** in CD_3CN.

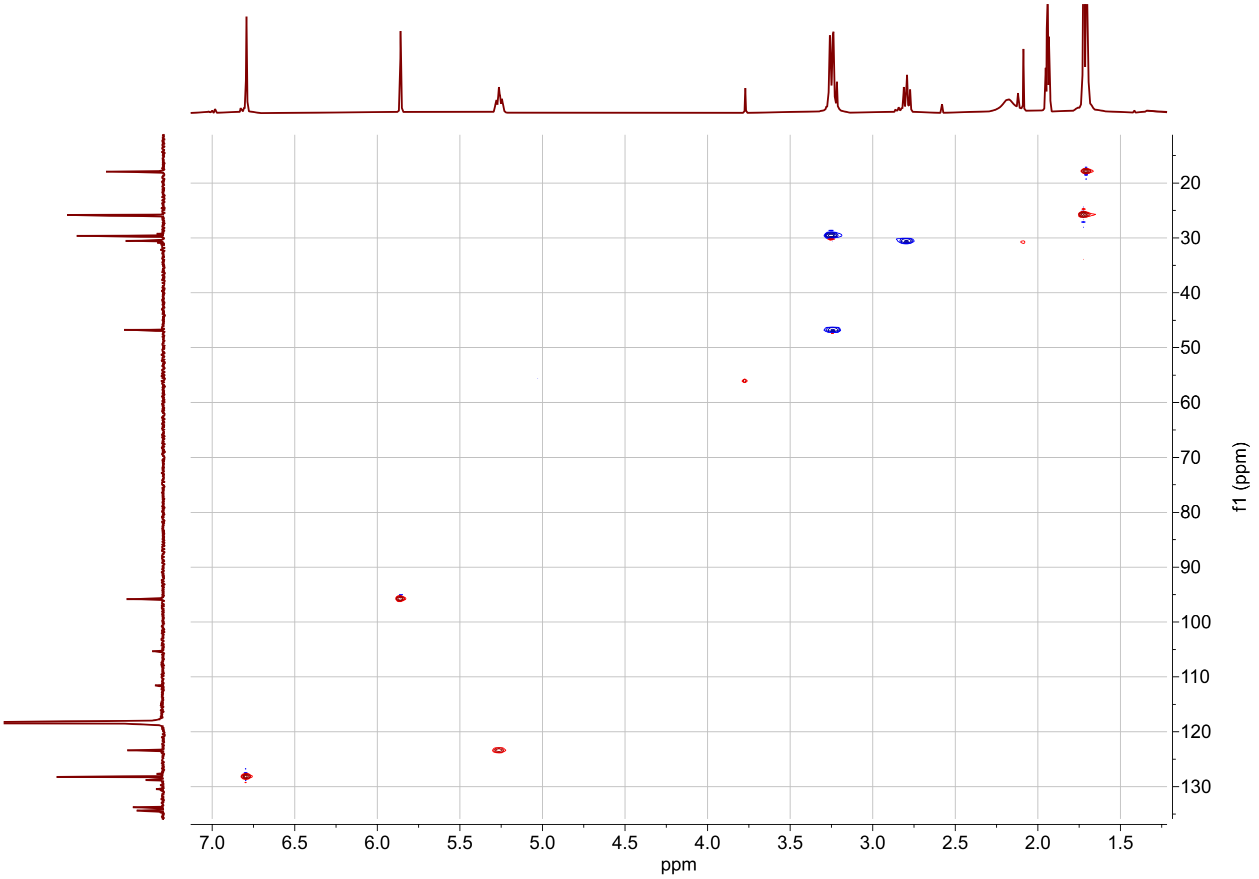

Figure S18. HMBC NMR Spectrum (400/101 MHz) of **3** in CD_3CN.

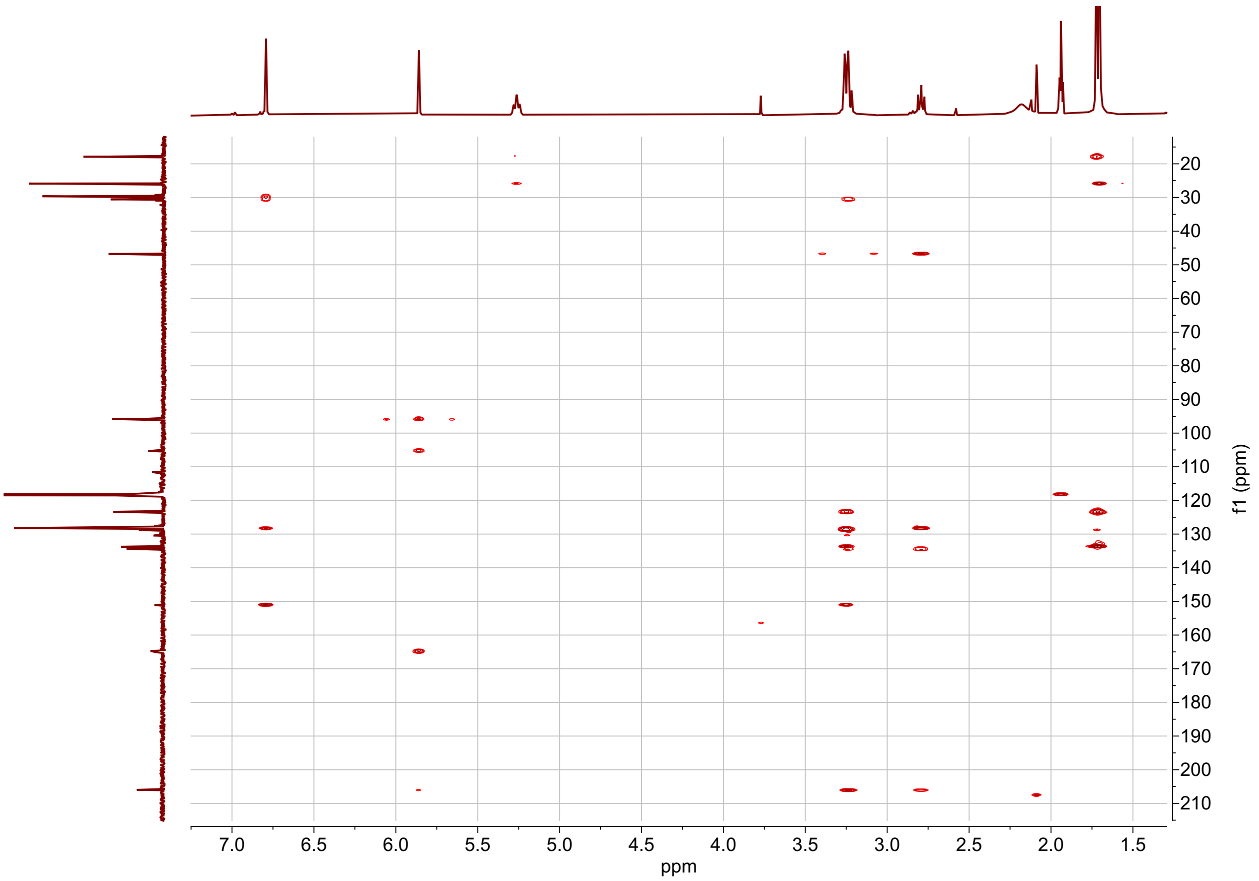

Figure S19. ^{1}H NMR Spectrum (400 MHz) of **4** in CD3OD.

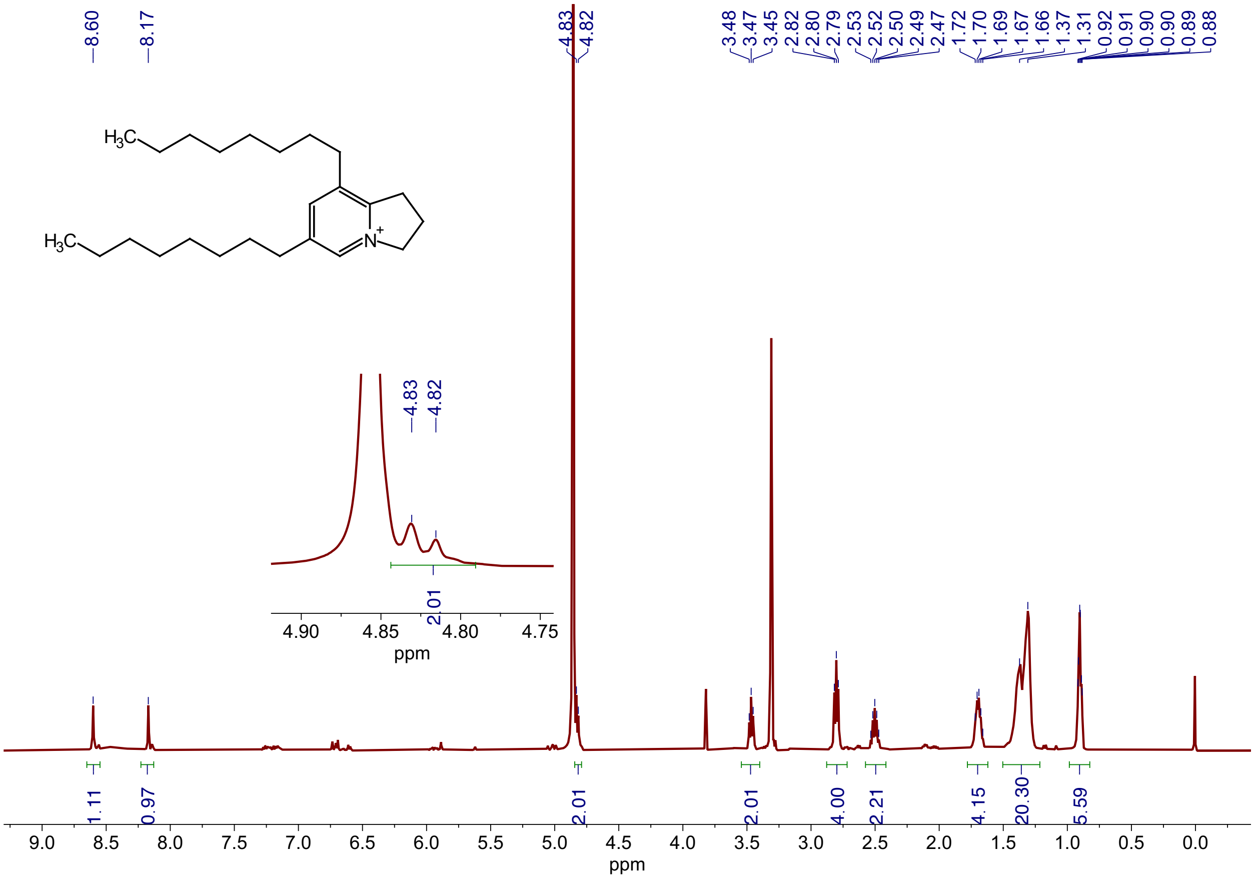

Figure S20. Expanded ¹H NMR Spectrum (400 MHz) of **4** in CD_3OD, highlighting the peaks in the 0–3.5 ppm region.

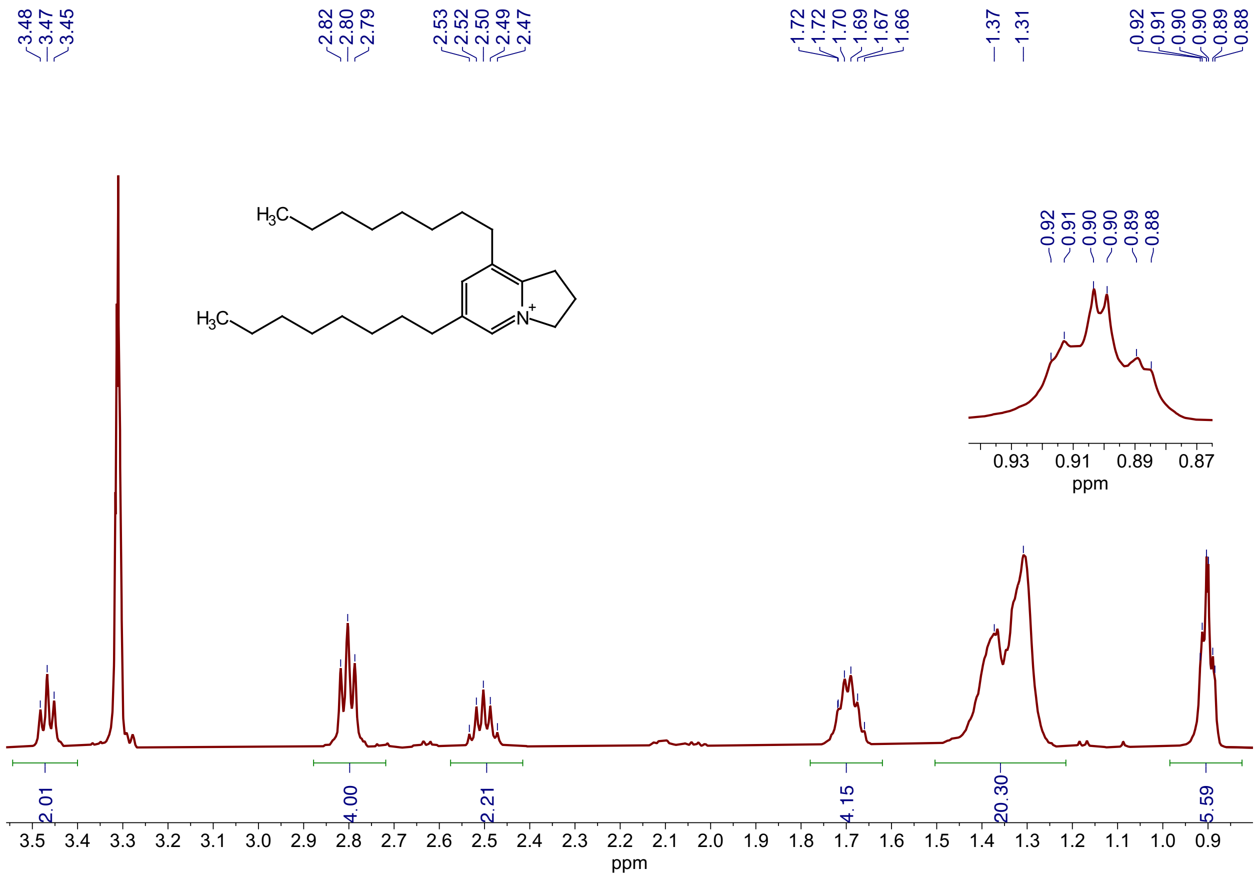

Figure S21. ^{13}C NMR Spectrum (101 MHz) of 4 in CD_3OD.

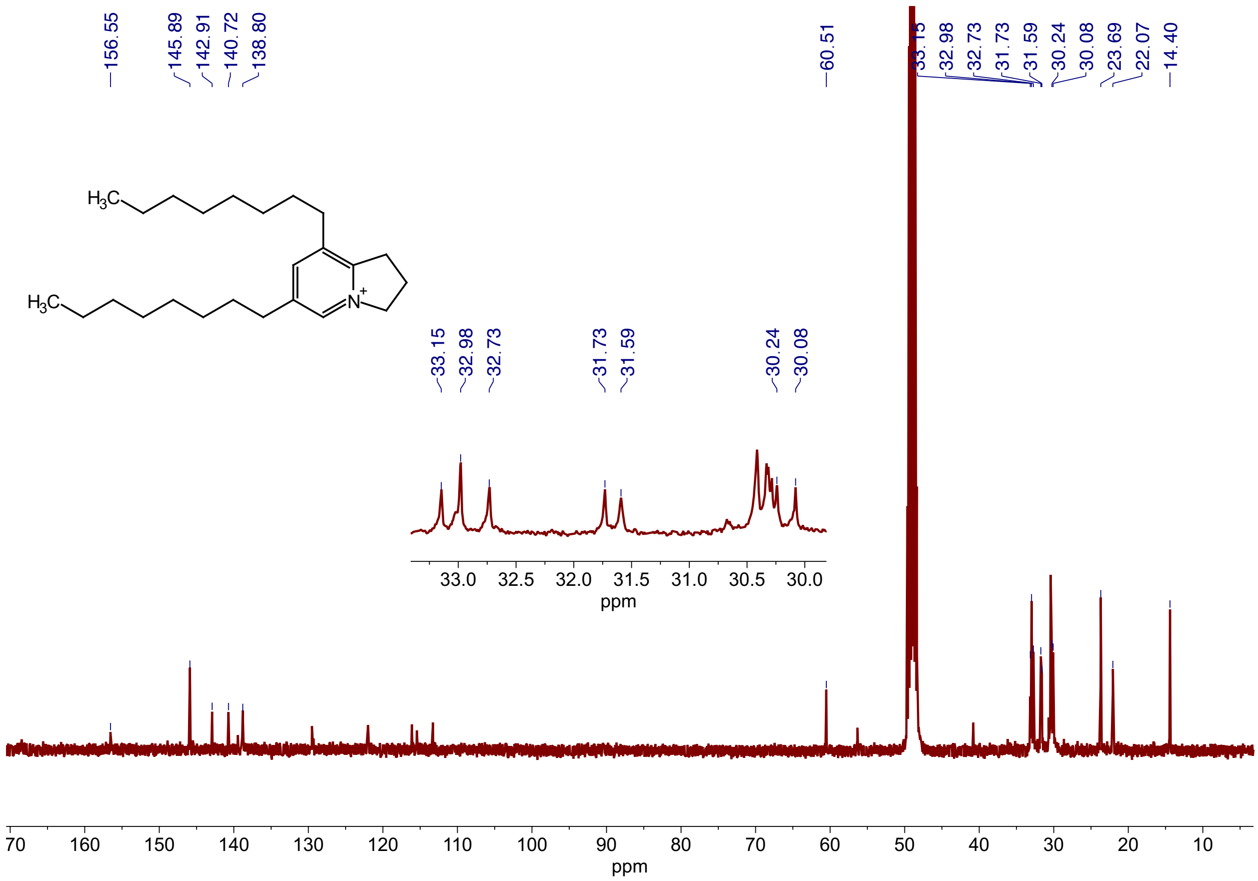

Figure S22. ^1H-^1H COSY NMR Spectrum (400 MHz) of **4** in CD_3OD.

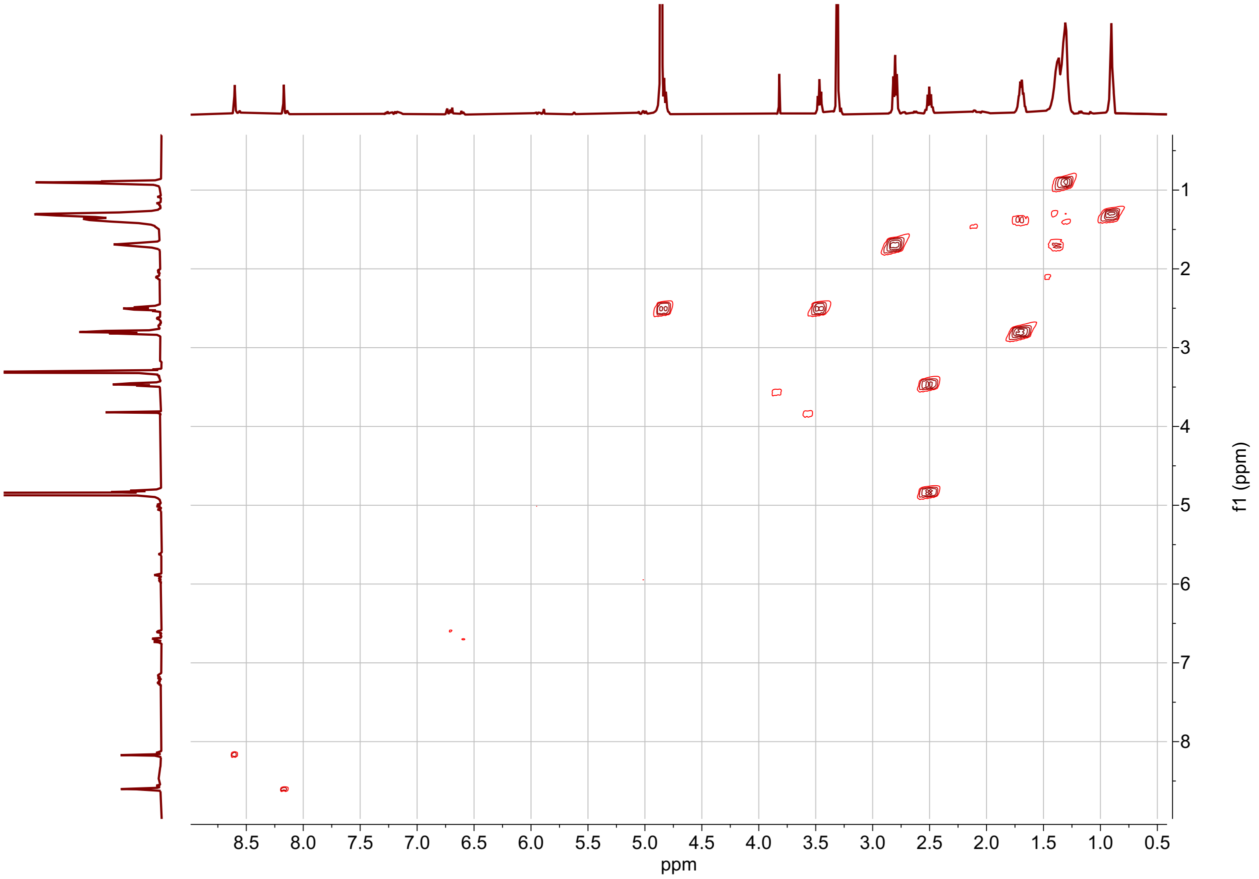

Figure S23. HSQC NMR Spectrum (400/101 MHz) of **4** in CD3OD.

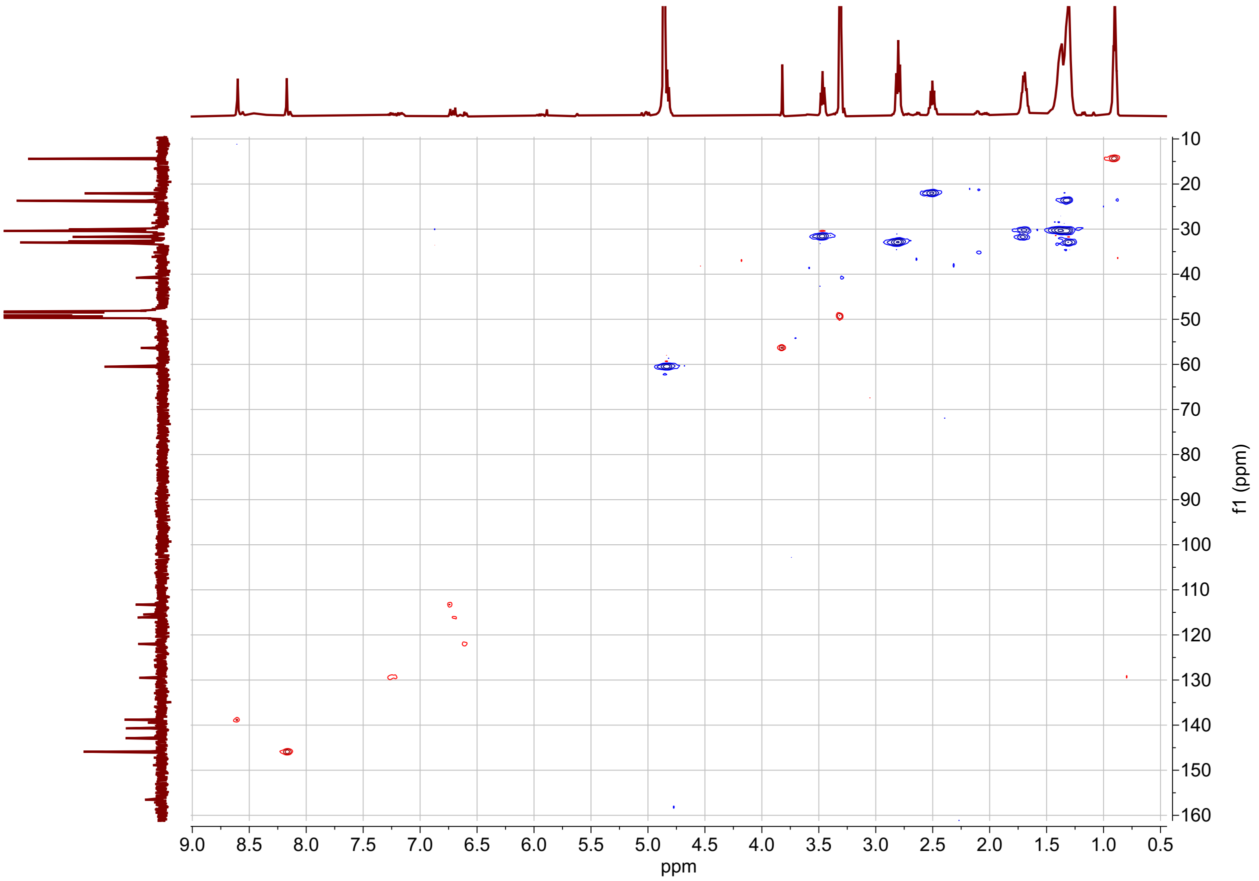

Figure S24. HMBC NMR Spectrum (400/101 MHz) of **4** in CD_3OD.

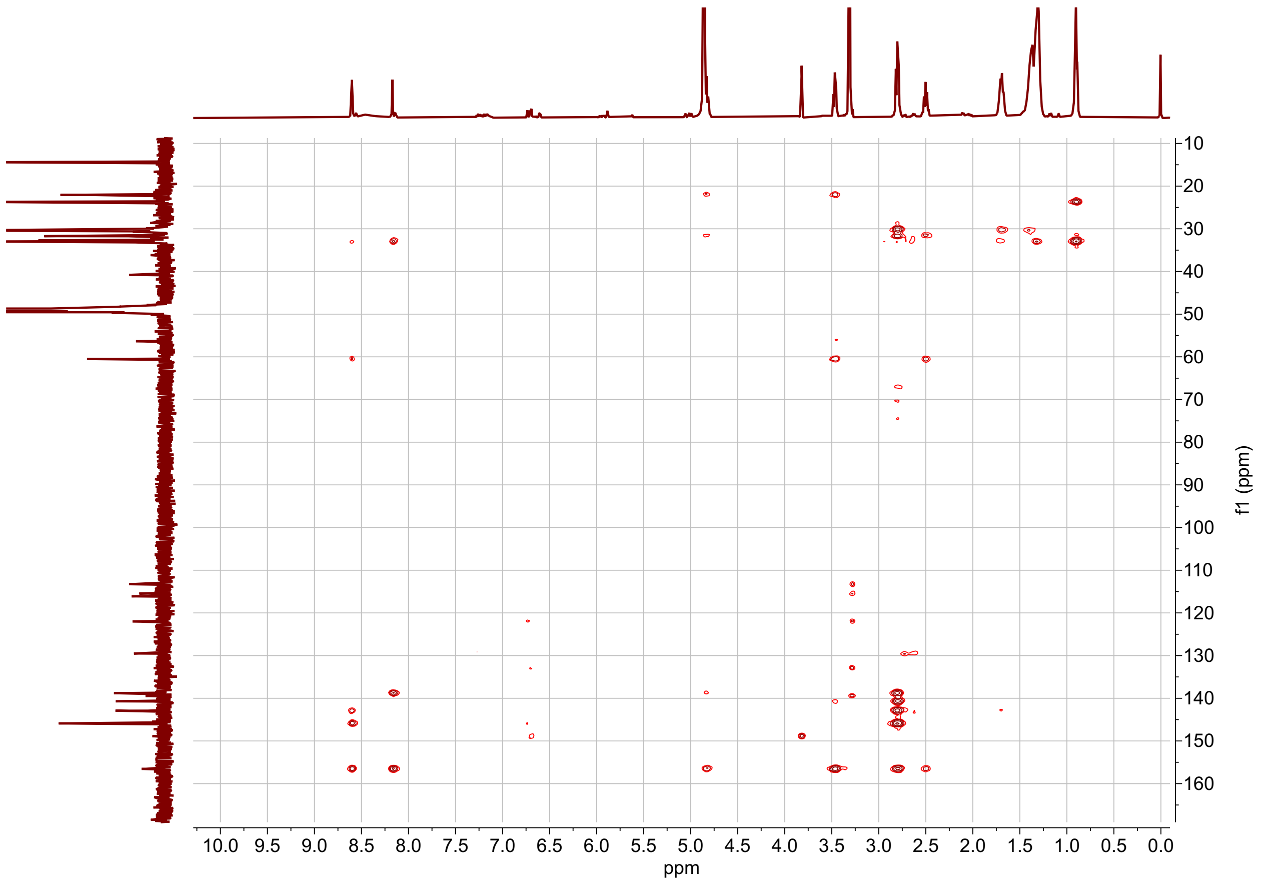

Figure S25. ^{1}H NMR Spectrum (400 MHz) of Synthesized **4** in CD_3OD.

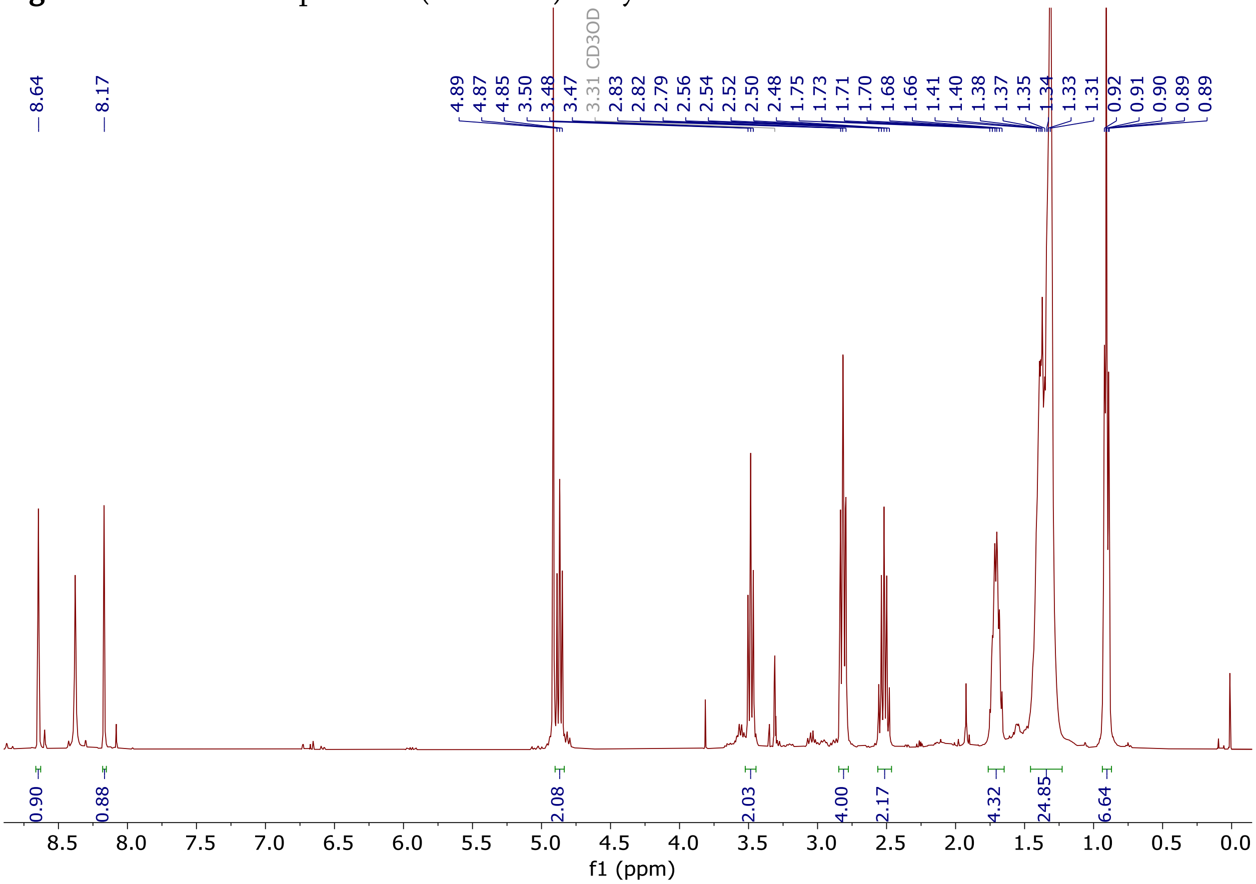

Figure S26. ^{13}C NMR Spectrum (101 MHz) of synthesized **4** in CD_3OD.

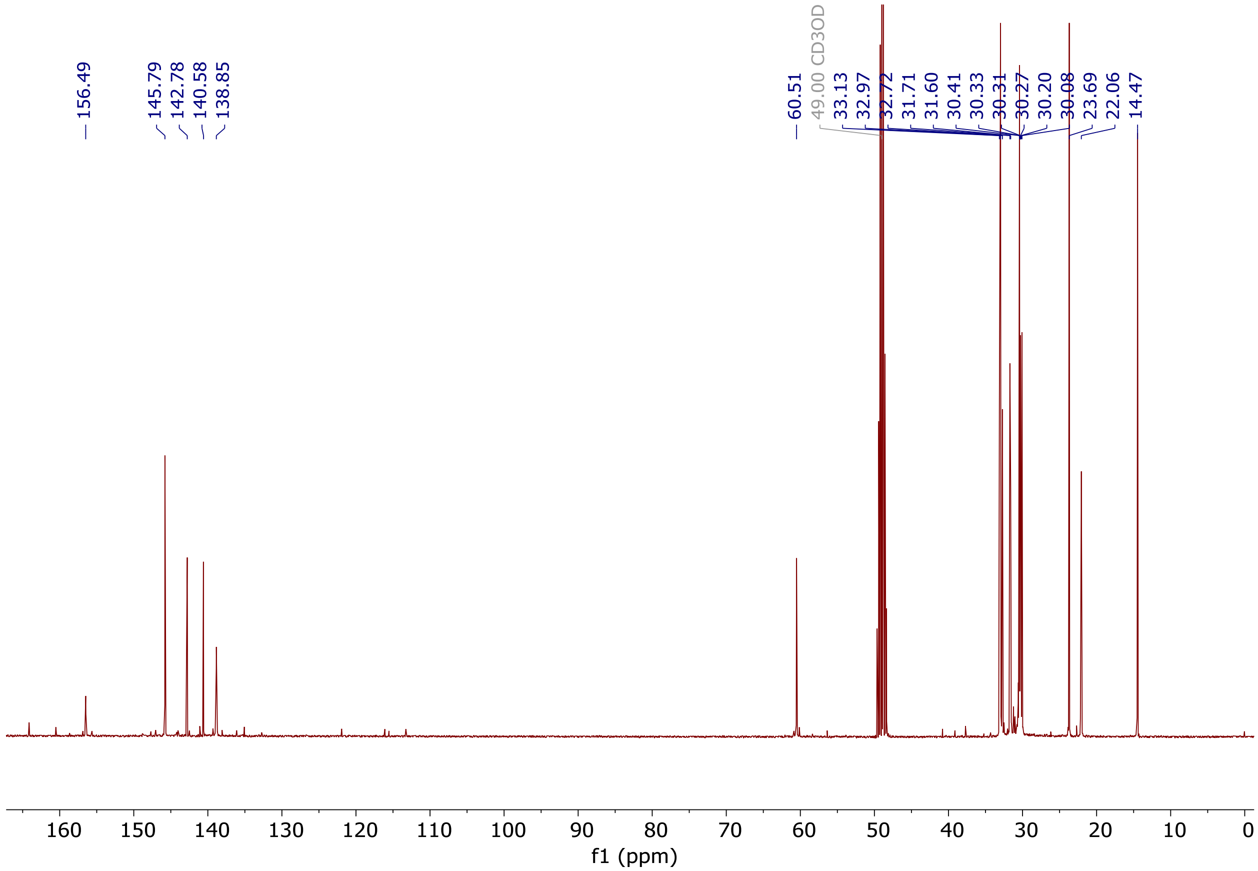

Figure S27. ^1H-^1H COSY NMR Spectrum (400 MHz) of synthesized **4** in CD_3OD.

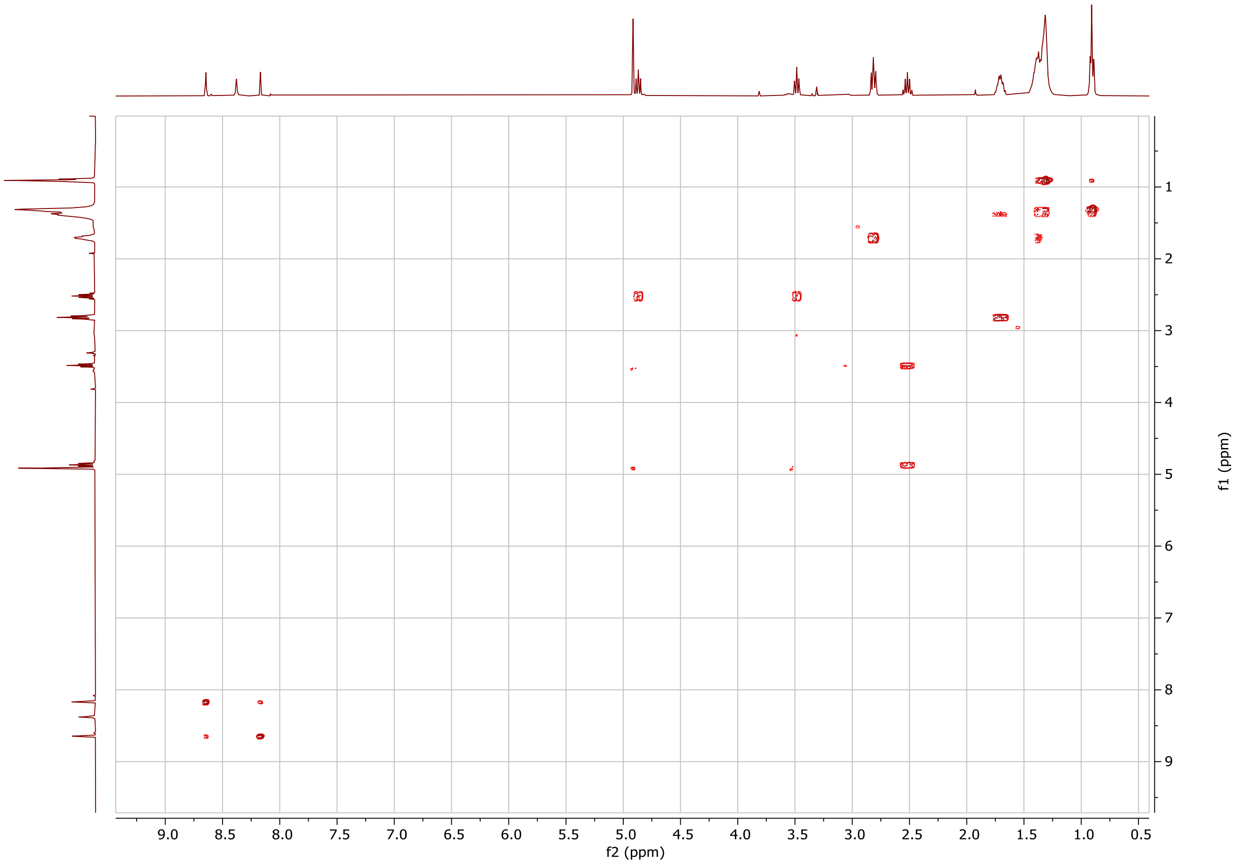

Figure S28. HSQC NMR Spectrum (400/101 MHz) of synthesized **4** in CD_3OD.

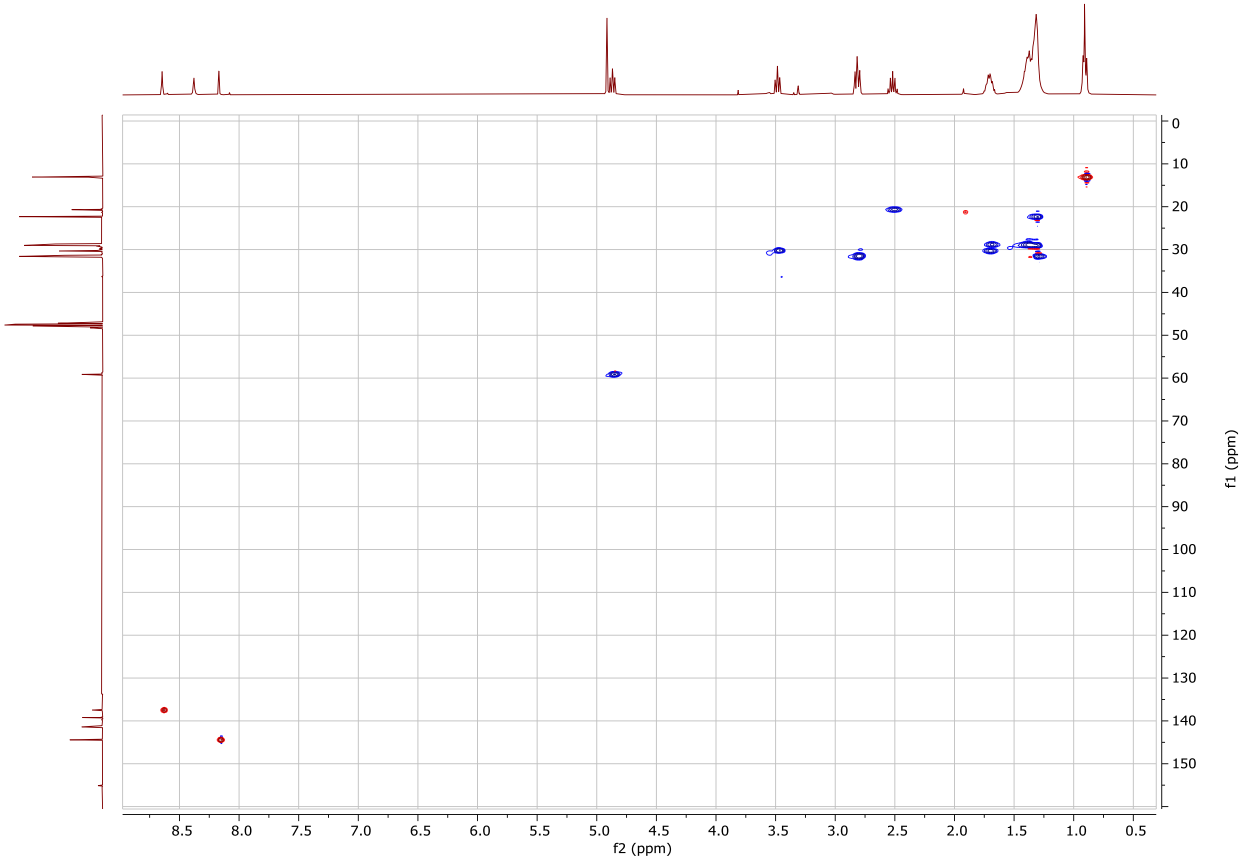

Figure S29. HMBC NMR Spectrum (400/101 MHz) of synthesized **4** in CD_3OD.

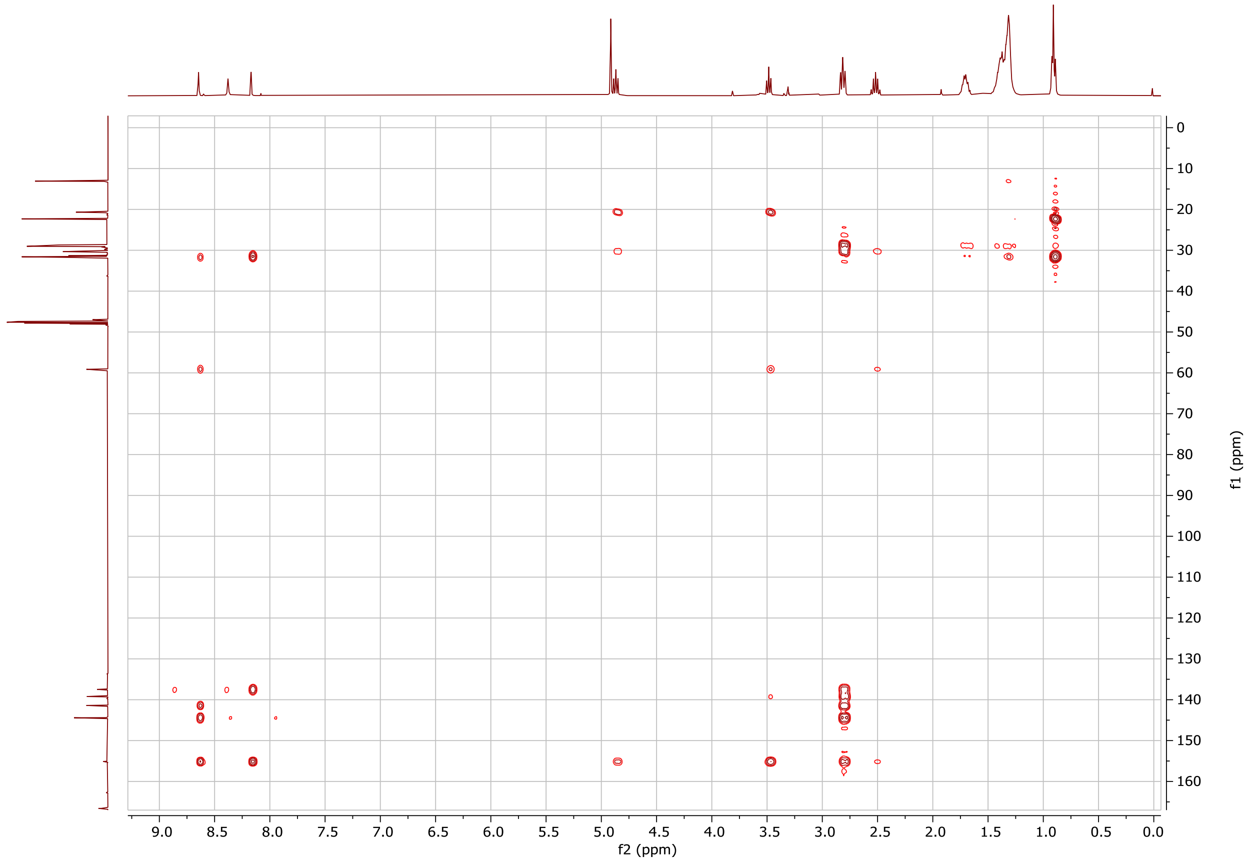

Figure S30.^{1}H NMR Spectrum (400 MHz) of **5** in $CDCl_3$.

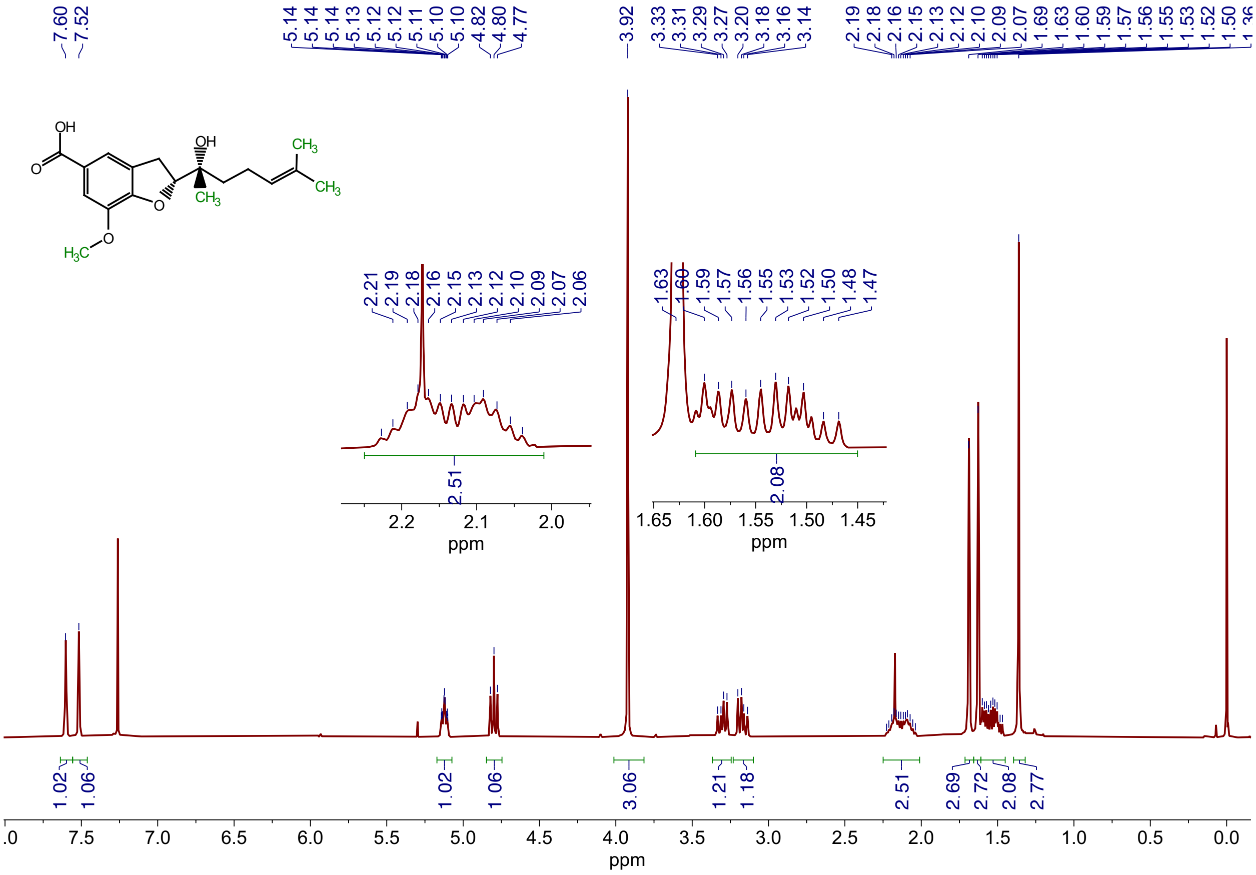

Figure S31. ^{13}C NMR Spectrum (101 MHz) of **5** in $CDCl_3$.

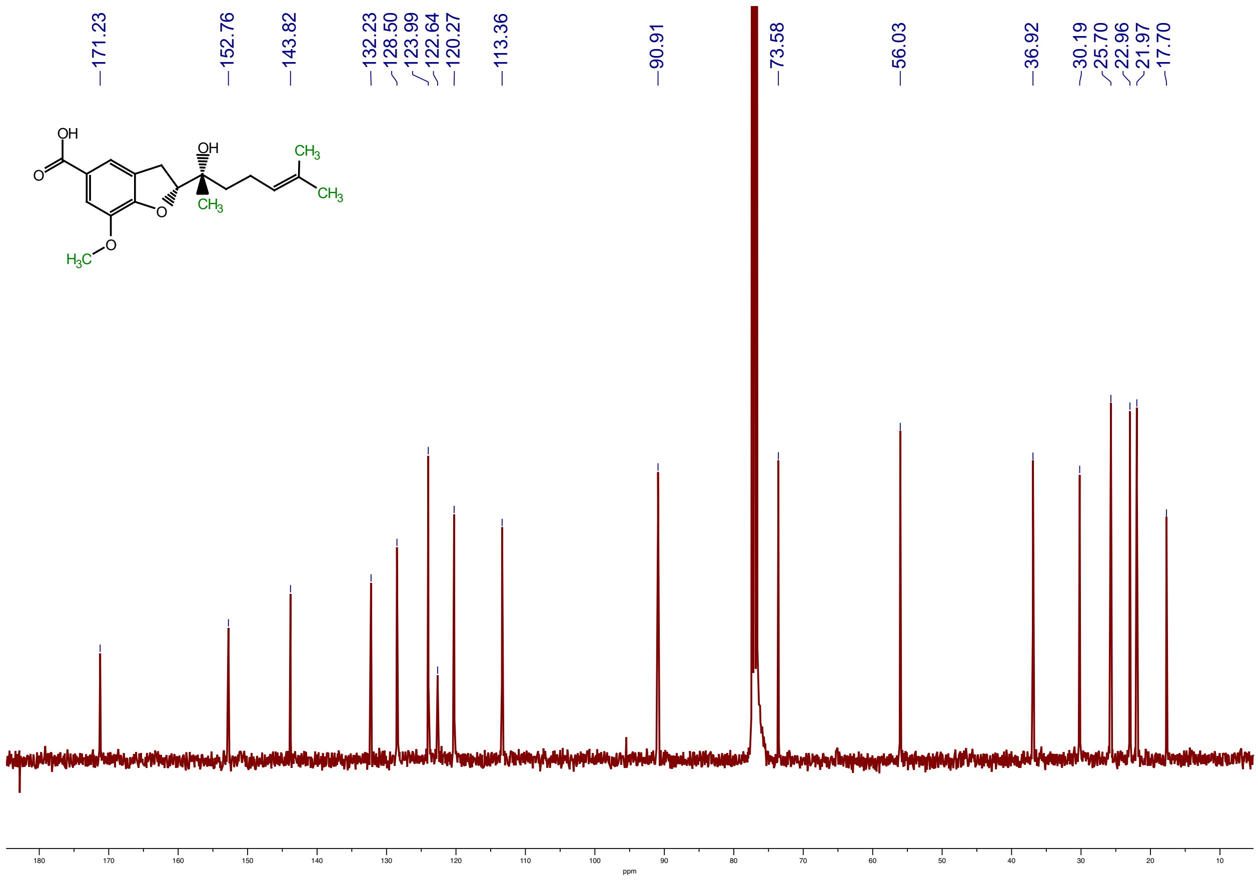

Supplement: Supplementary file 1 [file molecules-30-02020-s001.zip › molecules-3547700-supplementary-correct.pdf]
